# Chemical Constituents of *Hedyotis diffusa* and Their Anti-Inflammatory Bioactivities

**DOI:** 10.3390/antiox11020335

**Published:** 2022-02-09

**Authors:** Hsin-Yi Hung, Kun-Ching Cheng, Ping-Chung Kuo, I-Tsen Chen, Yue-Chiun Li, Tsong-Long Hwang, Sio-Hong Lam, Tian-Shung Wu

**Affiliations:** 1 School of Pharmacy, College of Medicine, National Cheng Kung University, Tainan 70101, Taiwan; z10308005@email.ncku.edu.tw (H.-Y.H.); z10502016@ncku.edu.tw (P.-C.K.); 10803048@gs.ncku.edu.tw (Y.-C.L.); 2 Taiwan Sugar Research Institute, Tainan 70176, Taiwan; a64128@taisugar.com.tw; 3 Department of Chemistry, National Cheng Kung University, Tainan 70101, Taiwan; l36021212@gs.ncku.edu.tw; 4 Graduate Institute of Natural Products, College of Medicine, Chang Gung University, Taoyuan 33305, Taiwan; htl@mail.cgu.edu.tw; 5 Research Center for Chinese Herbal Medicine, Research Center for Food and Cosmetic Safety, Graduate Institute of Health Industry Technology, College of Human Ecology, Chang Gung University of Science and Technology, Taoyuan 33305, Taiwan; 6 Department of Anesthesiology, Chang Gung Memorial Hospital, Taoyuan 33305, Taiwan

**Keywords:** *Hedyotis diffusa*, superoxide anion, elastase release, human neutrophils, anthraquinone

## Abstract

Seven new anthraquinones with rare 2-isopropyldihydrofuran (**1**–**3**) and 2,2-dimethylpyrano (**4**–**7**) moieties together with thirty-four known compounds were isolated from the extracts of whole *Hedyotis diffusa* plants. Their structures were elucidated and established by various spectroscopic and spectrometric analytical methods. Among these isolates, selected compounds were examined for their anti-inflammatory activity. The results showed that rare substituted anthraquinones displayed potent inhibitory activity with IC_50_ values ranging from 0.15 ± 0.01 to 5.52 ± 1.59 µM on the *N*-formyl-methionyl-leucyl-phenylalanine/cytochalasin B (fMLP/CB)-induced superoxide anion generation and elastase release cellular models. Meanwhile, the proposed drug target of the active anthraquinone was studied by computer modeling. The binding affinity between the anti-inflammatory anthraquinone and elastase was evaluated by molecular docking. These results provided the scientific insight into the medicinal values of *Hedyotis diffusa* and vision of development as lead compounds.

## 1. Introduction

*Hedyotis diffusa* Willd. (Rubiaceae) is a plant commonly used as folk medicine and distributed in southern provinces of China [1]. The herb of *H. diffusa* has the effects of clearing heat and detoxifying, relieving pain and dispelling masses, diuresis, and dehumidification [2]. It is mainly used for treating lung heat, asthma and cough, gastroenteritis, appendicitis, urinary system infection, throat swelling and pain, intestinal carbuncle sore, dysentery, malignant tumors, etc. [2]. According to the literature, more than 180 compounds have been characterized from *H. diffusa*, including iridoids, flavonoids, anthraquinones, phenolics, volatile oils, and polysaccharides [3,4,5,6,7,8]. Previous studies indicated that *H. diffusa* exhibits various pharmacological activities, including antioxidant, antiangiogenic, anti-inflammatory, hepatoprotective, apoptosis, and anticancer activities [3,8,9,10,11,12,13,14,15,16]. Ursolic and oleanolic acid isolated from *H. diffusa* display specific anti-tumor activity against colorectal cancer COLO205, liver cancer Hep 3B, and lung cancer H460 cell lines [17]. Moreover, methylanthraquinones from *H. diffusa* induced cell apoptosis by changing Fas and Fas Ligand (Fas/FasL) and activating caspase−8 in THP−1 cells of human leukemia [18] or increased Ca^2+^ concentration in human breast cancer cells through phosphorylation of c-Jun N-terminal kinase (JNK) protein kinase and activation of calpain to promote cell apoptosis [19]. 2-Hydroxy-3-methylanthraquinone can also enhance the apoptosis of leukemia cells U937 by modulating mitogen-activated protein kinase (MAPK), activating p-p38MAPK and reducing phosphorylated extracellular signal-regulated kinase (p-ERK1/2), with IC_50_ of 66 μM [20]. Iridoid glycoside *E*−6-*O*-*p*-methoxycinnamoyl scandoside methyl ester inhibited human neutrophil elastase with an IC_50_ of 18 μM [21]. Total flavonoids of *H. diffusa* improved the colonic mucosal damage, increased superoxide dismutase (SOD) activity in colon tissue, and reduced the myeloperoxidase, malondialdehyde (MDA), and nitric oxide (NO) activities in ulcerative colitis model rats [22]. Treatment with a homogeneous polysaccharide (25, 100, and 200 μg/mL) from *H. diffusa* resulted in growth inhibitory effect on A549 cells by inducing apoptosis [23].

Neutrophils, the largest type of macrophages, account for 50 to 60% of the total circulating white blood cells and play a major role in inflammatory response [24]. In addition to phagocytosis and enzyme secretion against pathogenic bacteria, neutrophils also secrete some peroxides, such as superoxide anion. Moreover, neutrophils are involved in other immune responses during inflammation, such as the production of elastase. The main function of elastase is to hydrolyze elastin, which can decompose injured cells and invading pathogenic bacteria in the infected area, and to complete the entire protection of host cells and tissues through apoptosis [25,26]. Therefore, inhibition of elastase secretion and superoxide anion formation can effectively reduce cell inflammation. A human neutrophil cell model activated by *N*-formyl-methionyl-leucyl-phenylalanine/cytochalasin B (fMLP/CB) to inhibit superoxide anion generation and elastase release was used as a screening platform for anti-inflammatory activity in our previous study [27,28]. In the preliminary screening of a series of Chinese herbal medicines with heat-clearing and detoxification potentials, the *H. diffusa* ethanol extracts displayed 71.71 and 38.26% inhibitory activities at 10 μg/mL in fMLP/CB-induced superoxide anion generation and elastase release assay, respectively. Moreover, the ethanol extract of *H. diffusa* also has an inhibitory effect on hepatitis C virus and Dengue virus, with IC_50_ and EC_50_ values against hepatitis C virus of 131.1 and 49.5 µg/mL, respectively (unpublished data). At a sample concentration of 25 μg/mL, the methanol extract of *H. diffusa* reduced the RNA expression of Dengue virus by 30.0 ± 8.1% (unpublished data). According to these experimental screening data, the chemical components of the ethanol extract of *H. diffusa* were thoroughly separated and identified. The anti-inflammatory activity of the major chemical components and their molecular docking with elastase were also investigated. This research is expected to provide an important reference for the development of anti-inflammatory lead compounds, healthy foods, and cosmetic products.

## 2. Materials and Methods

### 2.1. General Experimental Procedures

A Jasco P–2000 digital polarimeter (Jasco, Tokyo, Japan) with 589 nm filter was used to measure the optical rotations of purified compounds. The ultraviolet (UV) spectra were determined on a Hitachi U–0080-D UV/Vis spectrometer (Hitachi, Tokyo, Japan) with a 1.0 cm length cell. The infrared (IR) spectra were recorded on a PerkinElmer FT-IF spectrum RX I (PerkinElmer, Waltham, MA, USA) using KBr pellets. Circular dichroism (CD) spectra were determined on the Jasco J–720 spectropolarimeter (Jasco, Tokyo, Japan). One-dimensional and two-dimensional NMR spectra were recorded on the Bruker Avance III 400 or Avance III HD 700 NMR spectrometer (Bruker, Billerica, MA, USA) using CDCl_3_, acetone-*d*_6_, or methanol-*d*_4_ as solvent with tetramethylsilane as an internal standard. HR-ESI mass spectra were acquired from the Bruker APEX II mass spectrometer. Preparative high performance liquid chromatography (HPLC) was carried out on a Shimadzu LC–8A instrument (Shimadzu, Kyoto, Japan) equipped with UV-VIS detector (Shimadzu SPD–10A, Kyoto, Japan) and a Cosmosil 5C18-MS-II column (20 × 250 mm, Nacalai Tesque Kyoto, Japan). Column chromatography was performed on Geduran Si 60 (40–63 μm, Merck, Darmstadt, Germany). Thin-layer chromatography (TLC) was carried out using precoated Kieselgel 60 F_254_ plates (Merck), in which compounds were visualized by UV light or spraying with anisaldehyde solution followed by heating at 120 °C.

### 2.2. Plant Material

Dried whole herbs of *H. diffusa* were purchased from Chuang Song Zong Pharmaceutical Co. Ltd., Pingtung, Taiwan, in September 2013. The plant materials were authenticated by Prof. Chang-Sheng Kuoh, Department of Life Science, National Cheng Kung University (NCKU), Tainan, Taiwan. A voucher specimen (TSWu 2015–001–001) was deposited at School of Pharmacy, College of Medicine, National Cheng Kung University, Tainan, Taiwan.

### 2.3. Extraction and Isolation

The whole herbs of *H. diffusa* (5.2 kg) were refluxed with 95% ethanol (3 × 10 L) to give ethanol extract (370 g) after evaporation under reduced pressure. This crude extract was suspended in water and partitioned successively with ethyl acetate (EtOAc) to afford the EtOAc soluble fraction (110 g), water soluble layer (220 g), and precipitate (40 g).

**Scheme 1:**
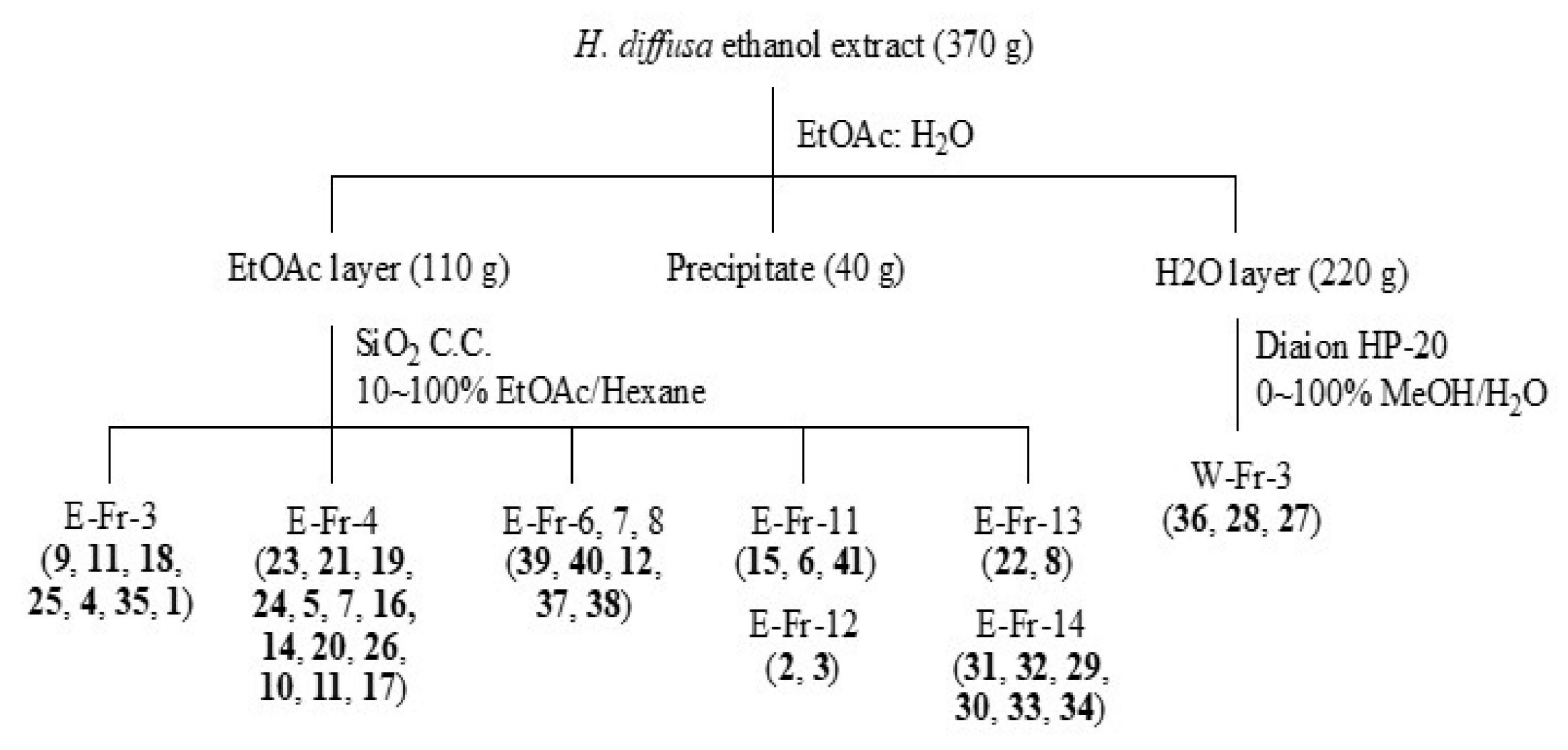
The extraction and isolation flow chart of *H. diffusa*.

The EtOAc-soluble fraction (110 g) was separated by silica gel column chromatography (SiO_2_ CC) eluted with a gradient of hexane-EtOAc (9:1 to 0:1) to give fifteen fractions (EtOAc-fractions, E-Fr. 1–15). E-Fr. 3 was isolated by SiO_2_ CC eluted with hexane–diisopropyl ether (30:1) to produce several subfractions, and recrystallization of subfractions E-Fr. 3–4 and 3–5 afforded **9** (1.9 mg), **11** (28.9 mg), **18** (8.2 mg), **25** (4.2 mg), **4** (9.1 mg), and **35** (2.2 mg). Compound **1** (2.4 mg) was further collected from E-Fr. 3–6 by preparative HPLC eluted with MeOH–H_2_O (9:1, flow rate = 10 mL/min). E-Fr. 4 was also chromatographed on SiO_2_ CC eluted with hexane–acetone (30:1), and further TLC purification or recrystallization of the resulting subfraction E-Fr. 4–3 afforded **23** (11.8 mg). Compounds **21** (17.8 mg), **19** (18.2 mg), **24** (1.0 mg), **5** (2.4 mg), and **7** (0.5 mg) were collected after separation by silica gel CC eluted with hexane–chloroform (1:2), preparative TLC, and recrystallization of subfraction E-Fr. 4–5. Subfraction E-Fr. 4–8 was isolated by SiO_2_ CC, eluted with hexane–di-isopropyl ether (1:1) to give **16** (1.4 mg), **14** (2.3 mg), **20** (3.5 mg), and **26** (7.1 mg). Subfraction E-Fr. 4–9 was also isolated by the similar procedures described as Fr. 4–8, and finally **10** (0.6 mg), **11** (19.3 mg), and **17** (1.2 mg) were collected. Recrystallization of E-Fr. 6, 7, and 8 produced mixture of **39** and **40** (424.2 mg), **12** (566.4 mg), and mixture of **37** and **38** (120.6 mg), respectively. Fraction E-Fr. 11 was subjected to SiO_2_ CC eluted with diisopropyl ether–hexane (30:1) to afford several subfractions. Of these, selected subfractions were further isolated by SiO_2_ CC and subsequent preparative TLC to yield **15** (17.2 mg), **6** (0.6 mg), and **41** (14.8 mg). Compounds **2** (3.3 mg) and **3** (1.1 mg) were obtained from Fraction E-Fr. 12 by SiO_2_ CC eluted with chloroform–acetone (30:1). Fraction E-Fr. 13 was subjected to SiO_2_ CC eluted with chloroform–acetone (10:1) to produce **22** (4.6 mg) and **8** (2.7 mg). Fraction E-Fr. 14 was isolated by SiO_2_ CC eluted with chloroform–MeOH (20:1) and a step gradient of MeOH to afford several subfractions. Compounds **31** (174.2 mg) and **32** (174.3 mg) were collected from subfraction E-Fr. 14–4 after preparative HPLC eluted with MeOH–H_2_O (1:1, flow rate = 10 mL/min). The same procedure was undertaken to obtain **29** (30.8 mg) and **30** (30.4 mg) from subfraction E-Fr. 14–6. Subfraction E-Fr. 14–5 was separated by a preparative HPLC eluted with MeOH–H_2_O (4:6, flow rate = 10 mL/min) to collect **33** (27.3 mg) and **34** (27.3 mg). The water-soluble layer (220 g) was separated by Diaion HP−20 CC, eluted with a gradient of MeOH-H_2_O (0:1 to 1:0) to give eleven fractions (Water-fractions, W-Fr. 1–11). W-Fr. 7 was separated by SiO_2_ CC eluted with chloroform–MeOH (8:1) to afford **36** (2.8 mg). Compounds **27** (14.2 mg) and **28** (70.1 mg) were isolated from W-Fr. 11 by SiO_2_ CC eluted with chloroform–MeOH (5:1) (summarized in Scheme 1).

### 2.4. Spectral and Physical Data of **1**–**7**

#### 2.4.1. Diffusaquinone A (**1**)

Yellow powder; [α]*_D_*^25^ + 2.4 (*c* 0.1, MeOH); UV (MeOH) *λ*_max_ 375, 274, 245, 211 nm; IR (KBr) *ν*_max_ 2927, 2861, 1668, 1585, 1447, 1293 cm^−1^; ECD (CHCl_3_) nm (Mol. CD) 321 (+0.01), 298 (+1.58), 267 (+0.19), 255 (+0.42), 240 (−1.19), 227 (+1.01), 213 (−0.89); ^1^H NMR (400 MHz, CDCl_3_), see Table 1; ^13^C NMR (100 MHz, CDCl_3_), see Table 2; ESI-MS *m/z* 305 [M+H]^+^; HR-ESI-MS *m/z* 305.1174 [M+H]^+^ (calcd. for C_20_H_17_O_3_, 305.1172).

#### 2.4.2. Diffusaquinone B (**2**)

Yellow powder; [α]*_D_*^25^ − 4.6 (*c* 0.1, MeOH); UV (MeOH) *λ*_max_ 413, 293, 248, 210 nm; IR (KBr) *ν*_max_ 3389, 2927,2857, 1664, 1574, 1344, 1292 cm^−1^; ECD (MeOH) nm (Mol. CD) 350 (+0.17), 342 (0.00), 306 (+1.36), 280 (−0.25), 244 (+0.24), 208 (−1.07), 197 (+0.03); ^1^H NMR (400 MHz, acetone-d_6_), see Table 1; ^13^C NMR (100 MHz, acetone-d_6_), see Table 2; ESI-MS *m/z* 339 [M+H]^+^; HR-ESI-MS *m/z* 361.1045 [M+Na]^+^ (calcd. for C_20_H_18_O_5_Na, 361.1046).

#### 2.4.3. Diffusaquinone C (**3**)

Yellow powder; [α]*_D_*^25^ + 119.5 (*c* 0.1, MeOH); UV (MeOH) *λ*_max_ 400, 291, 249, 211 nm; IR (KBr) *ν*_max_ 3445, 2927, 1662, 1574, 1348, 1292 cm^−1^; ECD (MeOH) nm (Mol. CD) 368 (−0.32), 351 (+0.25), 342 (+0.01), 303 (+1.08), 281 (−0.28), 229 (+0.33), 209 (−0.95); ^1^H NMR (400 MHz, methanol-d_4_), see Table 1; ^13^C NMR (100 MHz, methanol-d_4_), see Table 2; ESI-MS *m/z* 353 [M-H]^−^; HR-ESI-MS *m/z* 353.1033 [M-H]^−^ (calcd. for C_20_H_17_O_6_, 353.1031).

#### 2.4.4. Diffusaquinone D (**4**)

Yellow powder; UV (MeOH) *λ*_max_ 289, 276, 248, 208 nm; IR (KBr) *ν*_max_ 2926, 1727, 1665, 1578, 1327, 1304 cm^−1^; ^1^H NMR (400 MHz, CDCl_3_), see Table 1; ^13^C NMR (100 MHz, CDCl_3_), see Table 2; ESI-MS *m/z* 305 [M+H]^+^; HR-ESI-MS *m/z* 305.1170 [M+H]^+^ (calcd. for C_20_H_17_O_3_, 305.1172).

#### 2.4.5. Diffusaquinone E (**5**)

Yellow powder; UV (MeOH) *λ*_max_ 290, 273, 253, 208 nm; IR (KBr) *ν*_max_ 3377, 2978, 2930, 1665, 1571, 1333 cm^−1^; ^1^H NMR (400 MHz, CDCl_3_), see Table 1; ^13^C NMR (100 MHz, CDCl_3_), see Table 2; ESI-MS *m/z* 321 [M+H]^+^; HR-ESI-MS *m/z* 321.1124 [M+H]^+^ (calcd. for C_20_H_17_O_4_, 321.1121).

#### 2.4.6. Diffusaquinone F (**6**)

Yellow powder; UV (MeOH) *λ*_max_ 408, 287, 259, 214 nm; IR (KBr) *ν*_max_ 3300, 2925, 2855, 1700, 1663, 1560, 1361, 1332, 1303 cm^−1^; ^1^H NMR (700 MHz, methanol-d_4_), see Table 1; ^13^C NMR (175 MHz, methanol-d_4_), see Table 2; ESI-MS *m/z* 359 [M+Na]^+^; HR-ESI-MS *m/z* 359.0891 [M+Na]^+^ (calcd. for C_20_H_16_O_5_Na, 359.0890).

#### 2.4.7. Diffusaquinone G (**7**)

Yellow powder; UV (MeOH) *λ*_max_ 418, 294, 256, 223 nm; IR (KBr) *ν*_max_ 3393, 2931, 1642, 1566, 1353, 1320, 1253, 1119 cm^−1^; ^1^H NMR (400 MHz, CDCl_3_), see Table 1; ^13^C NMR (100 MHz, CDCl_3_), see Table 2; ESI-MS *m/z* 337 [M+H]^+^; HR-ESI-MS *m/z* 337.1068 [M+H]^+^ (calcd. for C_20_H_17_O_5_, 337.1071).

### 2.5. Anti-Inflammatory Bioactivity Examination

#### 2.5.1. Human Neutrophil Preparation

Neutrophils were isolated using the standard method of dextran sedimentation prior to centrifugation on a Ficoll Hypaque gradient and hypotonic lysis of erythrocytes. Blood was drawn from healthy human donors (20–30 years old) by venipuncture into heparin-coated vacutainer tubes; the protocol was approved by the review committee of Chang Gung Memorial Hospital (IRB plan number: 103–7405A3 and 201902217A3). Purified neutrophils were pelleted and then re-suspended in 1 mM calcium (Ca^2+^)-contained Hank’s balanced salt solution (HBSS) buffer at pH 7.4 for anti-inflammatory assays [27].

#### 2.5.2. Superoxide Anion Generation Measurement

The assay of the generation of superoxide anion was based on the SOD-inhibitable reduction of ferricytochrome c. Neutrophils (6 × 10^5^ cells/mL) were equilibrated in the presence of 0.6 mg/mL ferricytochrome *c* at 37 °C for 2 min and incubated with each test compound or vehicle (0.1% DMSO, negative control) for 5 min. Cells were incubated with cytochalasin B (CB, 1 μg/mL) for 3 min. Neutrophils were then activated by *N*-formyl-L-methionyl-L-leucyl-L-phenylalanine (fMLP, 100 nM). The changes in the absorbance of ferricytochrome *c* reduction at 550 nm were continuously monitored in a double-beam, six-cell positioner spectrophotometer (Hitachi U–3010, Tokyo, Japan) with constant stirring. A phosphatidylinositol 3-kinase (PIK3) inhibitor, LY294002, was used as a positive control [27].

#### 2.5.3. Elastase Release Assay

Degranulation of azurophilic granules was determined by elastase release as described previously. Elastase substrate used in experiments was MeO-Suc-Ala-Ala-Pro-Val-p-nitroanilide. After supplementation with MeO-Suc-Ala-Ala-Pro-Val-*p*-nitroanilide (100 µM), neutrophils (6 × 10^5^/mL) were equilibrated at 37 °C for 2 min and incubated with test compounds or vehicle (0.1% DMSO, negative control) for 5 min. Cells were activated by 100 nM fMLP and 0.5 μg/mL CB, and absorbance changes at 405 nm were continuously monitored to measure release of elastase. LY294002 was used as a positive control [27].

#### 2.5.4. Statistical Analysis

The results are expressed as the mean ± standard error of the mean (SEM). 50% Inhibition concentration (IC_50_) was calculated using a computer (PHARM/PCS v4.2). Student’s *t* test was used for statistical comparison among each group. Values of *p* less than 0.05 were considered statistically significant.

### 2.6. Molecular Docking Study

An AutoDock Vina software (v.1_1_2) was used for the in silico evaluation [29]. The crystal structure of the Human neutrophil elastase was downloaded from the Protein Databank (PDB ID: 1H1B). The 3D structures of ligands were constructed in the Chem3D program. AutodockTools (ADT v1.5.6) carries out the hydrogen supplementation, Gasteiger charge measurement of protein atoms, and selection of ligand flexible torsions. Center at 18.6, 11.8, and 22.8 (x, y, z) of grid box was determined. The binding affinity energy was provided as docking scores and shown in kcal/mol. Biovia Discovery Studio client 2020 analyzed the visualization of the best docking interactions [30].

## 3. Results and Discussion

The ethanol extract was fractionated into several layers by liquid–liquid partition. Further chromatography purification resulted in the characterization of twenty-six anthraquinones (**1**–**26**), eight iridoids glycosides (**27**–**34**), two phenolics (**35**–**36**), two triterpenoids (**37**–**38**), two steroids (**39**–**40**), and one amide (**41**). Among these isolates, seven anthraquinones, diffusaquinone A-H (**1**–**7**), were reported for the first time from natural sources, and their structures were established on the basis of 1D and 2D NMR and mass spectrometric analyses. All ^1^H and ^13^C NMR results are summarized in Table 1 and Table 2.

### 3.1. Structural Elucidation of Compounds **1**–**7**

**Figure 1:**
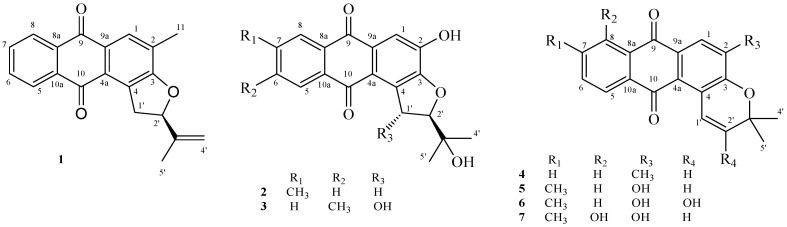
Structures of anthraquinones **1–7** isolated from *H. diffusa*.

**Figure 2:**
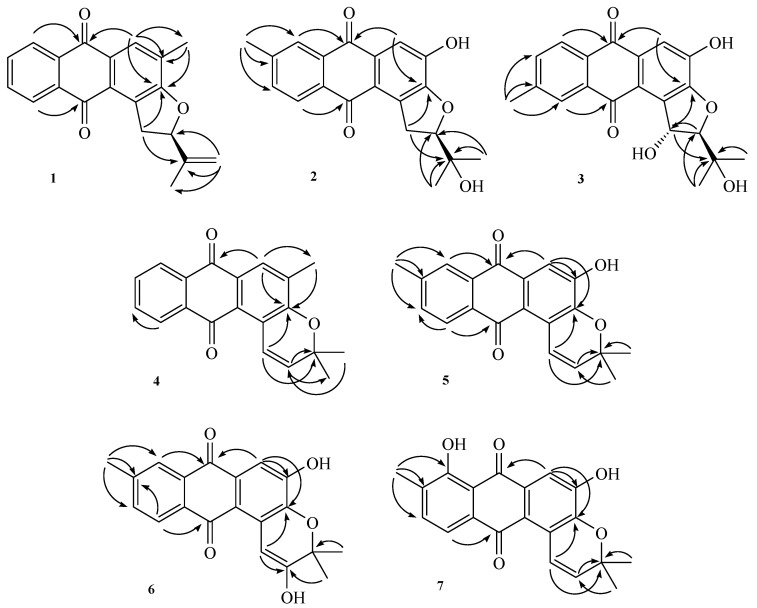
Significant HMBC correlations of anthraquinones **1**–**7**.

Compound **1** (Figure 1) was isolated as an optically active yellow powder. Molecular formula of C_20_H_16_O_3_ was established on the basis of HR-ESI-MS (*m/z* 305.1174 for [M+H]^+^, calcd. for C_20_H_17_O_3_, 305.1172, Figure S1). The absorbance maxima at 245 and 275 nm in its UV spectrum were the typical feature of an anthraquinone-type compound [31]. The IR spectrum showed absorption bands at 1668, 1585, and 1447 cm^−1^ that indicated the presence of conjugated carbonyl groups and aromatic ring functionalities. The ^1^H NMR spectrum of **1** (Table 1, Figure S2) exhibited signals for five aryl protons, composed of an ABCD system (δ 8.28 (1H, dd, *J* = 8.8, 2.4 Hz, H−8), 8.22 (1H, dd, *J* = 8.8, 2.4 Hz, H−5), 7.76 (2H, m, H−6/H−7)), and a singlet at δ 8.05 (1H, s, H−1). The ^13^C NMR spectra (Table 2, Figure S3) revealed twelve carbon signals corresponding to two aryl groups, composed of a di-substituted aryl (*δ* 127.2 (s, C−10a), 133.8 (s, C−8a), 127.1 (d, C−8), 133.9 (d, C−7), 133.5 (d, C−6) and 126.7 (d, C−5)) and a penta-substituted aryl (*δ* 127.2 (s, C−9a), 128.6 (s, C−4a), 128.1 (s, C−4), 164.3 (s, C−3), 125.8 (s, C−2), and 130.7 (d, C−1)). These signals together with two carbonyl carbons (*δ* 182.3 (C−9) and 184.5 (C−10)) and one methyl carbon (δ 15.8, Me−11) suggested the presence of a 9, 10-anthraquinone basic skeleton. The key heteronuclear multiple bond correlation (HMBC, Figure S4) ^2^*J*- and ^3^*J*-correlations of H−1 (δ 8.05) to C−2/C−3/C−9/C−11, H−5 (δ 8.22) to C−10, H−8 (δ 8.28) to C−9, Me−11 (δ 2.37) to C−2/C−3 determined the location of methyl group at C−2 therefore supported the 2-methyl−9, 10-anthraquinone skeleton. The NOESY correlations (Figure S5) between Me-11 to H-1 was also confirmed this connection. The remaining ^1^H NMR and COSY correlations (Figure S6) signals included those for an –OCH–CH_2_– system at δ 5.39 (1H, t, *J* = 8.0 Hz, H−2′), 3.93 (1H, dd, *J* = 18.0, 10.0 Hz, H−1′α), and 3.54 (1H, dd, *J* = 18.0, 8.0 Hz, H−1′β), together with one methyl signals (δ 1.80 (3H, s, Me−5′)) and two terminal olefinic protons (δ 5.13 (1H, br s, H−4′α), 4.96 (1H, br s, H−4′β)). In addition, five corresponding carbon signals in the ^13^C and HSQC NMR spectra (Figure S7), including two olefinic carbons (δ 143.4 (s, C−3′) and 112.4 (t, C−4′)), one oxygenated methine (δ 87.7, d, C−2′), one methylene (δ 36.1, t, C−1′), and one methyl (δ 17.1, q, C−5′) indicated the appearance of the isopentenyl dihydrofuranyl moiety in **1**. The observed ^2^*J*- and ^3^*J*- HMBC correlations (Figure 2 and Figure S4) of the isopentenyl moiety from H−1′α (1H, δ 3.93) to C−3, from H−1′β (1H, δ 3.54) to C−3′, and from H−4′β (δ 4.96, 1H, s) to C−3′/C−5′ established that this moiety was fused at C−3 and C−4 of the anthraquinone. The absolute configuration at C−2′ of **1** was determined by the CD spectrum (Figure S8), which showed a positive Cotton effect at 298 nm. This result is consistent with the positive value of *R*-dihydrocolumbianetin reported in the literature [32], which therefore determines the configuration of C−2′ as *R*. Based on these above data of **1**, its chemical structure was established as shown in Figure 1 and named trivially as diffusaquinone A.

Compounds **2** and **3** (Figure 1) were isolated as yellow solids and exhibited similar UV, IR, and ^1^H- and ^13^C-NMR spectra (Table 1 and Table 2) as those of diffusaquinone A (**1**). The molecular formula of **2** was determined as C_20_H_18_O_5_ by HR-ESI-MS data (*m/z* 361.1045 for [M+Na]^+^, Figure S9). In the aromatic region of ^1^H-NMR and COSY spectra of **2** (Table 1, Figures S10 and S11), an ABX system at δ 8.08 (1H, d, *J* = 8.0 Hz, H−5), δ 7.98 (1H, d, *J* = 1.6 Hz, H−8), and 7.65 (1H, dd, *J* = 8.0, 1.6 Hz, H−6) suggested the presence of a tri-substituted aryl group in **2**. The ^13^C-, DEPT and HSQC NMR spectra of **2** (Figures S12 and S13) revealed that the isopentenyl dihydrofuranyl moiety in **1** was replaced by five carbon signals at δ 92.0 (d, C−2′), 70.7 (s, C−3′), 32.2 (t, C−1′), 25.0 (q, C−5′), and 24.7(q, C−4′). Two singlet methyls at δ 1.27 (3H, s, Me−5) and δ 1.32 (3H, s, Me−4′) belonging to a 2-hydroxy-isopropylfuran moiety were located next to the oxygenated methine (δ_H_ 4.89 (1H, t, *J* = 8.0 Hz, H−2′); δ_C_ 92.0) evidenced by the HMBC correlations (Figure S14) from H−4′ (δ 1.32, 3H, s) and H−5′ (δ 1.27, 3H, s) to C−2′/C−3′, and from H−1′ (δ 3.70, 1H, d, *J* = 8.0 Hz) to C−3′. Another methyl group (δ 2.51, s, Me−11) and hydroxyl substituent were located at C−7 (δ 144.6, s) and C−2 (δ 145.7, s), as confirmed by the HMBC correlations from Me−11 (δ 2.51, 3H, s) to C−6/C−7/C−8, and from H−1 (δ 7.60, 1H, s) to C−3/C−9, respectively. From the NOESY spectrum (Figure S15) of **2**, the NOE correlations between H−1′ (δ 5.77, 1H, d, *J* = 4.0 Hz) and H−2′, and Me−4′/Me 5′ confirmed the relative configuration of H−1′ as β. The CD spectrum (Figure S16) of **2** also displayed a positive Cotton effect at 306 nm, which confirmed the same *R* configuration at C−2′ as that of **1**. Subsequently, all the other spectral data analyses confirmed the structure of **2** as 2-hydroxy−3,4-[2′-(1-hydroxy−1-methylethyl)-dihydrofurano]−7-methyl−9,10-anthraquinone, as shown in Figure 1, and named diffusaquinone B following the convention. The HR-ESI-MS analytical data (Figure S17) determined the molecular formula of **3** as C_20_H_18_O_6_. The ^1^H-, ^13^C-, COSY and HSQC NMR spectral (Figures S18–S21) characteristics revealed the possibility that **3** possessed one more hydroxyl group than **2**. Based on the ^2^*J*- and ^3^*J*-HMBC (Figure S22) correlations of Me−11 (δ 2.49, 3H, s) to C−5/C−6/C−7, the methyl group was determined to be attached at C−6 (δ 144.4, s) in **3** rather than at C−7 in **2**. The NOESY correlations (Figure S23) between Me-11 to H-5/H-7 was also confirmed this connection. The additional hydroxyl group was substituted at C−1′ (δ 73.3, d) of the dihydrofuran ring due to the ^3^*J*-HMBC correlations between H−1′ (δ 5.77, 1H, d, *J* = 4.0 Hz) and C−3/C−3′. The positive Cotton effect at 303 nm (Figure S24) displayed the same β-configuration of H−1′ as **2**. Therefore, the structure of **3** was concluded as 2-hydroxy−3,4-[1′-hydroxy−2′-(1-hydroxy−1-methylethyl)-dihydrofurano]−6-methyl−9,10-anthraquinone and named as diffusaquinone C.

Compound **4** (Figure 1), collected as yellow powder, had a molecular formula C_20_H_17_O_3_ deduced from HR-ESI-MS spectrum (*m/z* 305.1170 [M+H]^+^, calcd. 305.1172, Figure S25). Some of its 1D (Figures S26 and S27) and 2D NMR data (Figures S28–S31) were similar to those of **1**, including an ABCD system (δ 8.21 (2H, m, H−5/H−8), 7.76 (2H, m, H−6/H−7)) and a proton singlet at δ 8.07 (1H, s), suggesting the presence of 9,10-anthraquinone basic skeleton as that of **1** (Table 1 and Table 2). The methyl (δ 2.32, s, Me−11) was located at C−2 (δ 132.5, s), determined by the HMBC correlation (Figure S31) between H−1 (δ 8.07, 1H, s) and C−11 (δ 16.5, q). In addition, the presence of two methyls (δ 1.50 (6H, s, Me−4′/5′)), and two *cis*-coupled olefinic protons (δ 7.87 (1H, d, *J* = 10.4 Hz, H−1′) and 5.94 (1H, d, *J* = 10.4 Hz, H−2′)) were observed. Meanwhile, two olefinic carbons (δ 133.8 (d, C−2′), δ 120.9 (d, C−1′)), one oxygenated quaternary carbon (δ 76.6, s, C−3′), and two methyls (δ 28.0, q, C−4′/C−5′) in the ^13^C-NMR spectrum (Figure S27) supported the appearance of 2,2-dimethylpyrano moiety in **4**. In its HMBC spectrum, ^2^*J*- and ^3^*J*-correlations from H−1′ (δ 7.87) to C−3/C3′, from H−2′ (δ 5.94) to C−3′/C−4′/C−5′, and from Me−4′/Me−5′ (6H, δ 1.50, s) to C−2′ indicated that the 2,2-dimethylpyrano ring was fused at C−3/C−4, with the oxygen attached at C−3 (δ 157.1, s) in an angular form. All the other spectral data confirmed the structure of **4** as shown in Figure 1, and it was named diffusaquinone D.

Compound **5** (Figure 1) had a molecular formula C_20_H_17_O_4_ deduced from HR-ESI-MS analytical data (Figure S32), which had one more oxygen than **4**, revealed the possibility that **5** possessed one more hydroxyl group than **4**. The ^1^H and COSY NMR spectral data (Figures S33 and S34) in the aromatic region closely resembled those of **4**, except for the replacement of an ABCD system in **4** by an aryl ABX system in **5**. Summarizing its ^13^C NMR spectra (Figure S35), HSQC (Figure S36), HMBC (Figure S37) and NOESY (Figure S38) correlations proved that the core skeleton of 2-hydroxy−7-methyl−9,10-anthraquinone of **5** was the same as that of **2**. The remaining signals resulting from the 2,2-dimethylpyrano ring moiety at δ 1.53 (6H, s, Me−4′/5′), 7.93 (1H, d, *J* = 10.8 Hz, H−1′), 5.93 (1H, d, *J* = 10.8 Hz, H−2′) were also observed. The fusion position of 2,2-dimethylpyrano ring was confirmed at C−3 (δ 148.8, s)/C−4 (δ 132.0, s) by the ^2^*J*- and ^3^*J*-correlations from H−1′ (δ 7.93) to C−3 (δ 144.8, s)/C−3′ (δ 78.0, s), from H−2′ (δ 5.93) to C−3′, and from Me−4′/Me−5′ (δ 1.53) to C−3′ in the HMBC spectrum of **5**. Consequently, the structure of **5** was elucidated as shown in Figure 1 and named diffusaquinone E.

Compounds **6** and **7** (Figure 1) had the same molecular formula C_20_H_16_O_5_, determined according to the HR-ESI-MS analysis (Figures S39 and S40). After careful inspection of the 1D and 2D NMR spectral data (Figures S41–S46), we discovered one significant variation in **6,** namely that H−2′ in **5** had disappeared and instead one hydroxyl group substituted at δ 170.0 (C−2′, s) was observed. The location of hydroxy group was determined at C−2′ through the HMBC correlations (Figure S46) of H−1′ (δ 7.50, 1H, s) to C−3 (δ 148.8, s)/C−2′, and H−4′/5′ (δ 1.60, 6H, s) to C−2′. The ^13^C NMR data of **6** were assigned as shown in Table 1 based on the HMBC analytical results, and were elucidated as 2′-hydroxy diffusaquinone E and named diffusaquinone F. In 1D and 2D NMR (Figures S47–S52) of compound **7**, a significant broad singlet at δ 12.93 (brs, OH) indicated a hydroxyl group located next to the carbonyl group, leading to the formation of an intramolecular hydrogen bond (Figure S47). Additionally, the aryl ABX system in **6** was replaced by an AB system (δ 7.64 (1H, d, *J* = 7.6 Hz, H−5) and 7.48 (1H, d, *J* = 7.6 Hz, H−6)) in **7**. Its HMBC spectrum (Figure S51) showed correlations from Me−11 (δ 7.64, s) to C−6 (δ 136.9, d)/C−7 (δ 133.7, s)/C−8 (δ 160.4, s) and from H−1 (δ 7.80, s) to C−2 (δ 149.0, s)/C−3 (δ 145.2, s)/C−9 (δ 188.2, s). Combined, these data establish the core skeleton of **7** as 2,8-dihydroxy−7-methyl−9,10-anthraquinone. The remaining proton signals based on a 2,2-dimethylpyrano unit, consisting of two methyls (δ 1.54 (6H, s, Me−4′/5′)) and two *cis*-coupled olefinic protons (δ 7.92 (1H, d, *J* = 10.4 Hz, H−1′) and δ 5.94 (1H, d, *J* = 10.4 Hz, H−2′)), were determined to be fused at C−3 (δ 145.2, s) and C−4 (δ 122.1, s) by HMBC (Figure S51) and NOESY correlations (Figure S52). This result was further supported by the HMBC correlations, as shown in Figure 2. In conclusion, **7** was characterized as 8-hydroxy diffusaquinone E and named diffusaquinone G.

### 3.2. Anti-Inflammatory Activity

In addition to the seven anthraquinones discussed above (**1–7**), thirty-four known constituents were identified, including 2-hydroxy−6-hydroxymethylanthraquinone (**8**), tectoquinone (**9**), 2-hydroxymethyl−9,10-anthraquinone (**10**), 2-formyl−9,10-anthraquinone (**11**), 2-hydroxy−3-methyl−9,10-anthraquinone (**12**), 2-methoxy−3-methyl−9,10-anthraquinone (**13**), digiferruginol (**14**), 2-hydroxy−3-hydroxymethyl−9,10-anthraquinone (**15**), 1-methylalizarin (**16**), 2,6-dihydroxy−3-methyl−9,10-anthraquinone (**17**), 1-hydroxy−2-methoxy−3-methyl−9,10-anthraquinone (**18**), 2-hydroxy−1-methoxy−3-methyl−9,10-anthraquinone (**19**), 3-hydroxy−2-methoxy−6-methyl−9,10-anthraquinone (**20**), 2,3-dimethoxy−6-methyl−9,10-anthraquinone (**21**), 3-hydroxy−2-methoxy−6-hydroxymethyl−9,10-anthraquinone (**22**), physcion (**23**), robustaquinone B (**24**), erythroglaucin (**25**), capitellataquinone D (**26**), deacetyl asperulosidic acid methyl ester (**27**), scandoside methyl ester (**28**), *E*−6-*O*-*p*-coumaroyl scandoside methyl ester (**29**), *Z*−6-*O*-*p*-coumaroyl scandoside methyl ester (**30**), *E*−6-*O*-*p*-methoxycinnamoyl scandoside methyl ester (**31**), *Z*−6-*O*-*p*-methoxycinnamoyl scandoside methyl ester (**32**), *E*−6-*O*-feruloyl scandoside methyl ester (**33**), *Z*−6-*O*-feruloyl scandoside methyl ester (**34**), 4,7-dimethoxy−5-methyl−1,3-benzodioxole (**35**), *p*-coumaric acid (**36**), mixture of ursolic acid (**37**) and oleanolic acid (**38**), mixture of stigmasterol (**39**) and β-sitosterol (**40**), and aurantiamide acetate (**41**) (references provided in the Supplementary Data, Appendix A).

Among the isolated constituents, twenty-eight compounds with structural diversity were subjected to the human neutrophil cellular model and examined for their inhibitory activity on superoxide anion generation and elastase release (Table 3). According to the bioactivity data, some anthraquinones exhibited significant anti-inflammatory activity, while other compounds, such as iridoid glycosides (**27**–**34**), steroids (**39**–**40**), and amide (**41**), are not active. Compounds **1**, **2**, and **26**, anthraquinones with a 2-isopropyldihydrofuran moiety, displayed the most potent anti-inflammatory activity with the IC_50_ values ranged from 0.92 to 1.71 μM for superoxide anion generation and from 0.71 to 2.40 μM for elastase release. Compounds **4** and **5** with a 2,2-dimethylpyrano ring moiety also showed the potent anti-inflammatory activity with the IC_50_ values of 5.52 ± 1.59 and 0.15 ± 0.01 μM for inhibition of superoxide anion generation, and 3.25 ± 0.80 and 0.20 ± 0.02 μM for inhibition of elastase release, respectively. However, the substituents of tricyclic 9, 10-anthraquinones and their structure–activity relationships are still unclear, and further studies of pharmacological mechanisms and the binding receptor are required. In addition, our unpublished experimental data indicate that the ethanol extract of *H. diffusa* also had a moderate inhibitory effect on hepatitis C virus and Dengue virus. Whether these antiviral effects were related to the anti-inflammatory effects of these compounds remains to be further explored.

### 3.3. Molecular Docking Study

**Figure 3:**
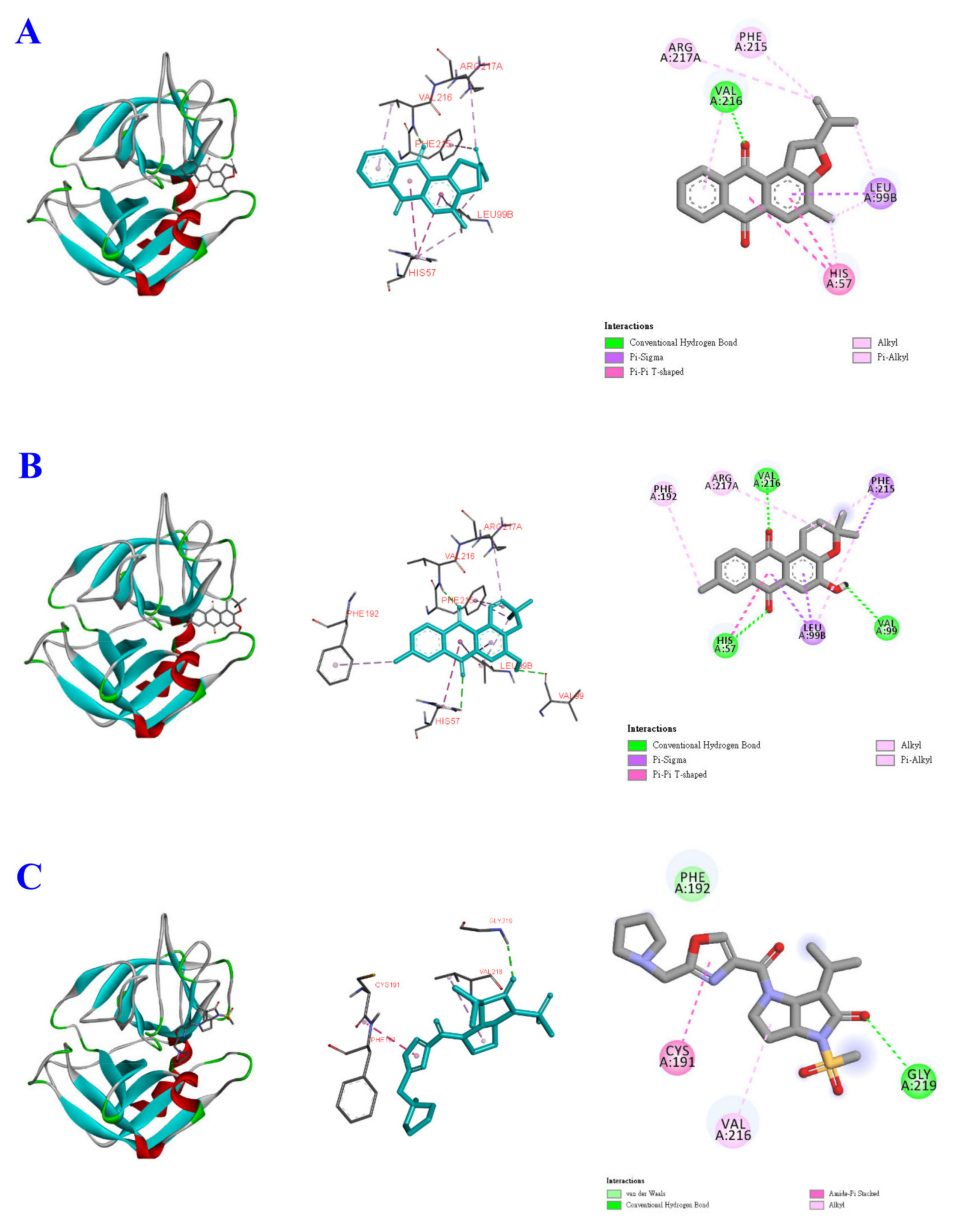
In silico modeling of (**A**) **1**, (**B**) **5**, and (**C**) GW475151 docking into the human neutrophil elastase receptor.

Molecular docking is a popular computing technique that can accurately predict the conformation and affinity between the ligand and the active pocket [33,34]. The docking method provides the high-dimensional space for possible interactions and evaluates the ranking of candidates based on a scoring function [35]. To illustrate the binding ability between anthraquinones and human neutrophil elastase, compounds **1**, **5**, and GW475151 were selected for molecular docking studies based on the above-mentioned experimental results. The binding affinity is shown in kcal/mol according to the computing results, and the calculated complex with the lowest energy was designed as the best docking configuration. GW475151 is an elastase inhibitor and is used as a native binding ligand [36]. It binds to elastase via Gly219 by hydrogen bond, and other amino acid residues Val216 and Cys191 by alkyl and amide-π-stacked interactions, and van der Waals (Figure 3). A stable complex is formed with binding energy of −6.0 kcal/mol (Table 4). Compounds **1** and **5** display even lower binding energy, and it indicates that they connect to proteins easier than GW475151 (Table 4). Several interactions are completed between **1** and elastase, including hydrogen bonds between Val216 and A-ring and carbonyl group of **1**; moreover, other interactions π-sigma, π-π T-shaped, alkyl, π-alkyl are linked to elastase by Leu99B, His57, Arg217A, and Phe215. **5** is bound with His57, Val99, and Val216 through hydrogen bond and linked with Phe215, Leu99B, Arg217A, and Phe192 via π-sigma, π-π T-shaped, alkyl, and π-alkyl effects, respectively. These interactions promote **5** and elastase to establish a stable unit, therefore resulting in a significant binding affinity with the receptor. All these computing results coincide well with the experimental data of the bioactivity examination. Therefore, it is speculated that the inhibition of elastase release may be related to the binding of anthraquinones with elastase, and further pharmacological mechanisms need to be verified.

## 4. Conclusions

In summary, a total of forty-one compounds, including seven new anthraquinones, were isolated from EtOAc layer of the ethanolic extract of *Hedyotis diffusa*. Among all the isolated compounds, anthraquinones and iridoids glycosides were the main components. Some anthraquinones, especially with 2-isopropyldihydrofuran or 2,2-dimethylpyrano moiety, showed promising anti-inflammatory activities for inhibiting superoxide anion generation and elastase release. Compound **1** with a 2-isopropyldihydrofuran moiety displayed the most potent anti-inflammatory activity for superoxide anion generation and elastase release with IC_50_ values 0.92 ± 0.22 μM and 0.71 ± 0.22 μM, respectively. Compound **5** containing the 2,2-dimethylpyrano ring moiety inhibited superoxide anions generation and elastase release with the IC_50_ values of 0.15 ± 0.01 μM and 0.20 ± 0.02 μM, respectively. These additional functional groups can significantly improve the anti-inflammatory effects of anthraquinones. The calculation results of molecular docking also confirmed the good binding affinity of **1** and **5** with neutrophil elastase. These functional groups are usually observed in natural products such as coumarins, acridones, and flavonoids but are rarely found in the anthraquinone skeleton. Such substructures can be used as a reference for medicinal chemistry derivation of anti-inflammatory lead compounds and as new candidates for the adjuvant therapy in the future.

## Figures and Tables

**Table 1:** The ^1^H NMR spectral data of compounds **1**–**7**.

	1 ^a^	2 ^b^	3 ^c^	4 ^a^	5 ^a^	6 ^d^	7 ^a^
Position	δ_H_ (ppm, multi, *J* in Hz)						
1	8.05 (s)	7.60 (s)	7.45 (s)	8.07 (s)	7.78 (s)	7.47 (s)	7.80 (s)
5	8.22 (dd, *J* = 8.8, 2.4 Hz)	8.08 (d, *J* = 8.0 Hz)	7.96 (brs)	8.21 (m)	8.10 (d, *J* = 8.0 Hz)	8.03 (d, *J* = 7.0 Hz)	7.64 (d, *J* = 7.6 Hz)
6	7.76 (m)	7.65 (dd, *J* = 8.0, 1.6 Hz)	-	7.73 (m)	7.55 (dd, *J* = 8.0, 1.2 Hz)	7.55 (brd, *J* = 7.0 Hz)	7.48 (d, *J* = 7.6 Hz)
7	7.76 (m)	-	7.58 (brd, *J* = 8.0 Hz)	7.73 (m)			
8	8.28 (dd, *J* = 8.8, 2.4 Hz)	7.98 (d, *J* = 1.6 Hz)	8.09 (d, *J* = 8.0 Hz)	8.21 (m)	8.01 (d, *J* = 1.2 Hz)	7.94 (brs)	
11	2.37 (s)	2.51 (s)	2.49 (s)	2.32 (s)	2.50 (s)	2.43 (s)	2.35 (s)
1′	3.93 (dd, *J* = 18.0, 10.0 Hz) α	3.70 (d, *J* = 8.0 Hz)	5.77 (d, *J* = 4.0 Hz)	7.87 (d, *J* = 10.4 Hz)	7.93 (d, *J* = 10.8 Hz)	7.50 (s)	7.92 (d, *J* = 10.4 Hz)
	3.54 (dd, *J* = 18.0, 8.0 Hz) ꞵ						
2′	5.39 (br t, *J* = 8.0 Hz)	4.89 (t, *J* = 8.0 Hz)	4.45 (d, *J* = 4.0 Hz)	5.94 (d, *J* = 10.4 Hz)	5.93 (d, *J* = 10.8 Hz)		5.94 (d, *J* = 10.4 Hz)
4′	5.13 (s) α	1.32 (s)	1.33 (s)	1.50 (s)	1.53 (s)	1.60 (s)	1.54 (s)
	4.96 (s) ꞵ						
5′	1.80 (s)	1.27 (s)	1.29 (s)	1.50 (s)	1.53 (s)	1.60 (s)	1.54 (s)
OH−8							12.93 (s)

^1^H NMR data (*δ*) were measured in ^a^ CDCl_3_ at 400 MHz; ^b^ acetone-d_6_ at 400 MHz; ^c^ methanol-d_4_ at 400 MHz; ^d^ methanol-d_4_ at 700 MHz.

**Table 2:** The ^13^C NMR spectral data of compounds **1**–**7**.

	1 ^a^	2 ^b^	3 ^c^	4 ^a^	5 ^a^	6 ^d^	7 ^a^
Position	δ_C_ (ppm)						
1	130.7	114.4	117.0	130.2	113.7	110.7	113.4
2	125.8	145.7	144.4	132.5	149.0	151.5	149.0
3	164.3	153.5	155.3	157.1	144.8	148.8	145.2
4	128.1	131.4	130.8	120.7	121.8	132.0	122.1
4a	128.6	128.9	129.9	126.6	121.8	120.1	122.1
5	126.7	126.6	126.5	127.0	127.2	127.9	118.9
6	133.5	134.3	144.4	133.6	134.6	136.0	136.9
7	133.9	144.6	134.0	133.4	144.3	146.0	133.7
8	127.1	126.7	126.5	126.5	126.8	128.3	160.4
8a	133.8	133.6	132.0	127.2	132.6	134.8	114.8
9	182.3	181.7	183.4	182.9	182.9	185.2	188.2
9a	127.2	123.4	120.0	132.9	129.7	134.0	129.2
10	184.5	182.6	183.3	185.7	184.6	184.2	184.1
10a	127.2	131.8	133.5	134.9	132.7	133.5	132.8
11	15.8	20.8	20.4	16.5	21.8	21.9	16.0
1′	36.1	32.2	73.3	120.9	120.9	104.1	120.8
2′	87.7	92.0	96.2	133.8	133.5	170.0	133.7
3′	143.4	70.7	70.7	76.6	78.0	70.2	78.2
4′	112.4	24.7	23.6	28.0	27.9	29.2	28.0
5′	17.1	25.0	24.1	28.0	27.9	29.2	28.0

^13^C NMR data (*δ*) were measured in ^a^ CDCl_3_ at 100 MHz; ^b^ acetone-d_6_ at 100 MHz; ^c^ methanol-d_4_ at 100 MHz; ^d^ methanol-d_4_ at 175 MHz.

**Table 3:** Effects of compounds on superoxide anion generation and elastase release in fMLP/CB-induced human neutrophils.

Compounds	Superoxide Anion		*p* Significance	Elastase Release		*p* Significance
	IC_50_ (µg/mL) ^b^	Inhibition% ^a^		IC_50_ (µg/mL) ^b^	Inhibition% ^a^	
1	0.92 ± 0.22	97.92 ± 4.14	***	0.71 ± 0.22	111.76 ± 2.97	***
2	1.71 ± 0.15	103.01 ± 0.74	***	2.40 ± 0.36	113.50 ± 5.04	***
4	5.52 ± 1.59	61.53 ± 5.76	***	3.25 ± 0.80	87.18 ± 2.92	***
5	0.15 ± 0.01	108.59 ± 1.66	***	0.20 ± 0.02	112.25 ± 4.99	***
8	3.55 ± 0.48	99.28 ± 0.29	***	3.88 ± 0.48	106.73 ± 3.10	***
9	1.58 ± 0.42	80.72 ± 3.25	***	>10	45.45 ± 6.31	**
11	5.29 ± 0.70	74.47 ± 2.80	***	>10	39.23 ± 6.61	**
12	>10	15.10 ± 5.84		>10	31.44 ± 4.62	**
13	>10	27.80 ± 4.16	**	>10	30.22 ± 4.05	**
14	>10	45.51 ± 6.33	**	>10	29.88 ± 4.62	**
15	2.30 ± 0.58	97.18 ± 0.65	***	5.12 ± 0.63	77.38 ± 2.92	***
18	>10	41.26 ± 6.15	***	>10	18.03 ± 6.74	*
19	4.96 ± 0.30	92.26 ± 2.51	***	5.68 ± 1.73	67.08 ± 6.09	***
20	3.66 ± 0.76	90.28 ± 6.56	***	3.94 ± 0.77	91.40 ± 2.14	***
21	>10	43.09 ± 6.56	**	>10	13.88 ± 3.52	*
22	>10	33.16 ± 5.19	***	>10	40.34 ± 5.97	**
23	>10	14.81 ± 6.93		>10	6.63 ± 1.27	**
25	>10	13.25 ± 5.20	*	>10	8.29 ± 6.57	
26	1.46 ± 0.29	100.83 ± 0.83	***	1.50 ± 0.25	113.90 ± 0.69	***
27	>10	2.57 ± 2.45		>10	−1.17 ± 2.02	
28	>10	13.05 ± 1.94	**	>10	9.17 ± 4.61	
29 + 30	>10	5.56 ± 0.49	***	>10	21.55 ± 3.17	**
31 + 32	>10	8.07 ± 3.06		>10	10.23 ± 2.80	*
33 + 34	>10	14.71 ± 2.44	**	>10	−1.47 ± 2.84	
35	0.90 ± 0.23	80.63 ± 1.22	***	5.15 ± 1.10	70.08 ± 2.36	***
37 + 38	0.41 ± 0.05	105.16 ± 1.41	***	1.81 ± 0.66	96.90 ± 5.09	***
39 + 40	>10	4.90 ± 4.15		>10	2.68 ± 3.00	
41	>10	16.75 ± 4.02	*	>10	21.21 ± 4.22	**
LY294002	0.75 ± 0.18	96.91 ± 5.99	***	1.26 ± 0.48	88.81 ± 1.59	***

^a^ Percentage of inhibition (Inh.%) at 10 µg/mL concentration. Results are presented as mean ± S.E.M. (*n* = 3–4). * *p* < 0.05, ** *p* < 0.01, *** *p* < 0.001 compared with the control value. ^b^ Concentration necessary for 50% inhibition (IC_50_).

**Table 4:** In silico computing binding energies of compounds **1**, **5**, and GW475151.

Ligands	Binding Affinity (kcal/mol)
**1**	−6.9
**5**	−7.2
GW475151	−5.8

## Data Availability

Data is contained within the article.

## Supplementary Materials

The following supporting information can be downloaded at https://www.mdpi.com/article/10.3390/antiox11020335/s1: Figures S1–S52: NMR and HRMS spectra of new compounds **1–7**.

## Appendix A

References for Known Compounds

2-hydroxy-6-hydroxymethylanthraquinone (**8**), *^1^*

tectoquinone (**9**), *^2^*

2-hydroxymethyl-9,10-anthraquinone (**10**), *^3^*

2-formyl-9,10-anthraquinone (**11**), *^4^*

2-hydroxy-3-methyl-9,10-anthraquinone (**12**), *^5^*

2-methoxy-3-methyl-9,10-anthraquinone (**13**), *^6^*

digiferruginol (**14**), *^7^*

2-hydroxy-3-hydroxymethyl-9,10-anthraquinone (**15**), *^8^*

1-methylalizarin (**16**), *^8^*

2,6-dihydroxy-3-methyl-9,10-anthraquinone (**17**), *^9^*

1-hydroxy-2-methoxy-3-methyl-9,10-anthraquinone (**18**), *^10^*

2-hydroxy-1-methoxy-3-methyl-9,10-anthraquinone (**19**), *^5^*

3-hydroxy-2-methoxy-6-methyl-9,10-anthraquinone (**20**), *^11^*

2,3-dimethoxy-6-methyl-9,10-anthraquinone (**21**), *^12^*

3-hydroxy-2-methoxy-6-hydroxymethyl-9,10-anthraquinone (**22**), *^13^*

physcion (**23**), *^14^*

robustaquinone B (**24**), *^15^*

erythroglaucin (**25**), *^16^*

capitellataquinone D (**26**), *^17^*

deacetyl asperulosidic acid methyl ester (**27**), *^18^*

scandoside methyl ester (**28**), *^18^*

*E*-6-*O*-*p*-coumaroyl scandoside methyl ester (**29**), *^19^*

*Z*-6-*O*-*p*-coumaroyl scandoside methyl ester (**30**), *^19^*

*E*-6-*O*-*p*-methoxycinnamoyl scandoside methyl ester (**31**), *^19^*

*Z*-6-*O*-*p*-methoxycinnamoyl scandoside methyl ester (**32**), *^19^*

*E*-6-*O*-feruloyl scandoside methyl ester (**33**), *^19^*

*Z*-6-*O*-feruloyl scandoside methyl ester (**34**), *^19^*

4,7-dimethoxy-5-methyl-1,3-benzodioxole (**35**), *^20^*

*p*-coumaric acid (**36**), *^21^*

mixture of ursolic acid (**37**) and oleanolic acid (**38**), *^22^*

mixture of stigmasterol (**39**) and ꞵ-sitosterol (**40**), *^23^*

aurantiamide acetate (**41**), *^24^*
