# Supplementary material for: Chemical Constituents of Hedyotis diffusa and Their Anti-Inflammatory Bioactivities
Source: Antioxidants (Basel). 2022 Feb 9;11(2):335. doi: 10.3390/antiox11020335 (PMC8868389; doi:10.3390/antiox11020335)
Supplement: Supplementary file 1 [file antioxidants-11-00335-s001.zip › antioxidants-1563413-supplementary.pdf]

Supplementary data

Chemical Constituents of *Hedyotis diffusa* and Their Anti-Inflammatory Bioactivities

Hsin-Yi Hung [1,†], Kun-Ching Cheng [2,†], Ping-Chung Kuo [1], I-Tsen Chen [3], Yue-Chiun Li [1], Tsong-Long Hwang [4,5,6], Sio-Hong Lam [1,*] and Tian-Shung Wu [1,*]

1 School of Pharmacy, College of Medicine, National Cheng Kung University, Tainan 70101, Taiwan; z10308005@email.ncku.edu.tw (H.-Y.H.); z10502016@ncku.edu.tw (P.-C.K.); 10803048@gs.ncku.edu.tw (Y.-C.L.)

2 Taiwan Sugar Research Institute, Tainan 70176, Taiwan; a64128@taisugar.com.tw

3 Department of Chemistry, National Cheng Kung University, Tainan 70101, Taiwan; l36021212@gs.ncku.edu.tw

4 Graduate Institute of Natural Products, College of Medicine, Chang Gung University, Taoyuan 33305, Taiwan; htl@mail.cgu.edu.tw

5 Research Center for Chinese Herbal Medicine, Research Center for Food and Cosmetic Safety, Graduate Institute of Health Industry Technology, College of Human Ecology, Chang Gung University of Science and Technology, Taoyuan 33305, Taiwan

6 Department of Anesthesiology, Chang Gung Memorial Hospital, Taoyuan 33305, Taiwan

* Correspondence: shlam@mail.ncku.edu.tw (S.-H.L.); tswu@mail.ncku.edu.tw (T.-S.W.); Tel.: +886–6–2353535 (ext. 6807) (S.-H.L.); Tel.: +886–6–2757575 (ext. 65333) (T.-S.W.)

† These authors contributed equally to this work.

Contents

Fig. S1. HRMS spectrum of **1**.

Fig. S2. ^{1}H NMR spectrum of **1** ($CDCl_3$, 400 MHz).

Fig. S3. ^{13}C and DEPT NMR spectrum of **1** ($CDCl_3$, 100 MHz).

Fig. S4. HMBC spectrum of **1** ($CDCl_3$, 400 MHz).

Fig. S5. NOESY spectrum of **1** ($CDCl_3$, 400 MHz).

Fig. S6. COSY spectrum of **1** ($CDCl_3$, 400 MHz).

Fig. S7. HSQC spectrum of **1** ($CDCl_3$, 400 MHz).

Fig. S8. CD spectrum of **1**.

Fig. S9. HRMS spectrum of **2**.

Fig. S10. ^{1}H NMR spectrum of **2** (Acetone-d_6, 400 MHz).

Fig. S11. COSY spectrum of **2** (Acetone-d_6, 400 MHz).

Fig. S12. ^{13}C and DEPT NMR spectrum of **2** (Acetone-d_6, 100 MHz).

Fig. S13. HSQC spectrum of **2** (Acetone-d_6, 400 MHz).

Fig. S14. HMBC spectrum of **2** (Acetone-d_6, 400 MHz).

Fig. S15. NOESY spectrum of **2** (Acetone-d_6, 400 MHz).

Fig. S16. CD spectrum of **2**.

Fig. S17. HRMS spectrum of **3**.

Fig. S18. ^{1}H NMR spectrum of **3** (CD_3OD, 400 MHz).

Fig. S19. ^{13}C and DEPT NMR spectrum of **3** (CD_3OD, 100 MHz).

Fig. S20. COSY spectrum of **3** (CD_3OD, 400 MHz).

Fig. S21. HSQC spectrum of **3** (CD_3OD, 400 MHz).

Fig. S22. HMBC spectrum of **3** (CD_3OD, 400 MHz).

Fig. S23. NOESY spectrum of **3** (CD_3OD, 400 MHz).

Fig. S24. CD spectrum of **3**.

Fig. S25. HRMS spectrum of **4**.

Fig. S26. ^{1}H NMR spectrum of **4** ($CDCl_3$, 400 MHz).

Fig. S27. ^{13}C and DEPT NMR spectrum of **4** ($CDCl_3$, 100 MHz).

Fig. S28. COSY spectrum of **4** ($CDCl_3$, 400 MHz).

Fig. S29. HSQC spectrum of **4** ($CDCl_3$, 400 MHz).

Fig. S30. NOESY spectrum of **4** ($CDCl_3$, 400 MHz).

Fig. S31. HMBC spectrum of **4** ($CDCl_3$, 400 MHz).

Fig. S32. HRMS spectrum of **5**.

Fig. S33. ^{1}H NMR spectrum of **5** ($CDCl_3$, 400 MHz).

Fig. S34. COSY spectrum of **5** ($CDCl_3$, 400 MHz).

Fig. S35. ^{13}C and DEPT NMR spectrum of **5** ($CDCl_3$, 400 MHz).

Fig. S36. HSQC spectrum of **5** ($CDCl_3$, 400 MHz).

Fig. S37. HMBC spectrum of **5** ($CDCl_3$, 400 MHz).

Fig. S38. NOESY spectrum of **5** ($CDCl_3$, 400 MHz).

Fig. S39. HRMS spectrum of **6**.

Fig. S40. HRMS spectrum of **7**.

Fig. S41. ^{1}H NMR spectrum of **6** (CD_3OD, 700 MHz).

Fig. S42. ^{13}C and DEPT NMR spectrum of **6** (CD_3OD, 175 MHz).

Fig. S43. COSY spectrum of **6** (CD_3OD, 700 MHz).

Fig. S44. HSQC spectrum of **6** (CD_3OD, 700 MHz).

Fig. S45. NOESY spectrum of **6** (CD_3OD, 700 MHz).

Fig. S46. HMBC spectrum of **6** (CD_3OD, 700 MHz).

Fig. S47. ^{1}H NMR spectrum of **7** ($CDCl_3$, 400 MHz).

Fig. S48. ^{13}C and DEPT NMR spectrum of **7** ($CDCl_3$, 400 MHz).

Fig. S49. COSY spectrum of **7** ($CDCl_3$, 400 MHz).

Fig. S50. HSQC spectrum of **7** ($CDCl_3$, 400 MHz).

Fig. S51. HMBC spectrum of **7** ($CDCl_3$, 400 MHz).

Fig. S52. NOESY spectrum of **7** ($CDCl_3$, 400 MHz).

Appendix A. References for known compounds **8-41**.

Figure S1. HRMS spectrum of **1**.

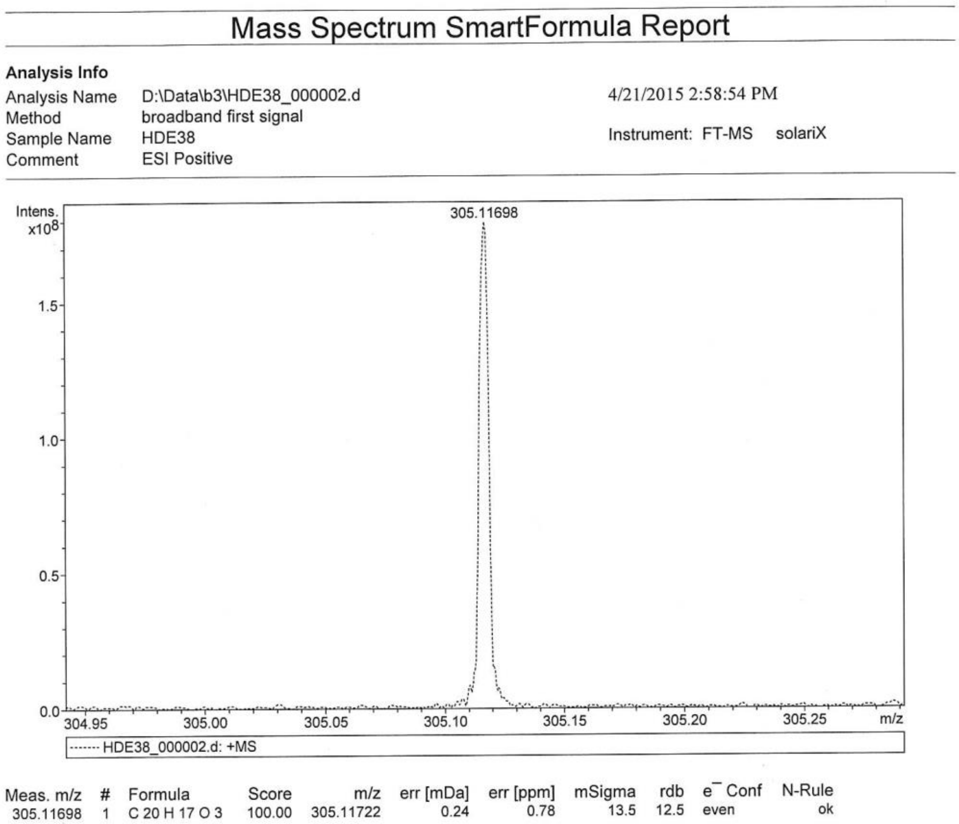

Figure S2. ^{1}H NMR spectrum of **1** ($CDCl_3$, 400 MHz).

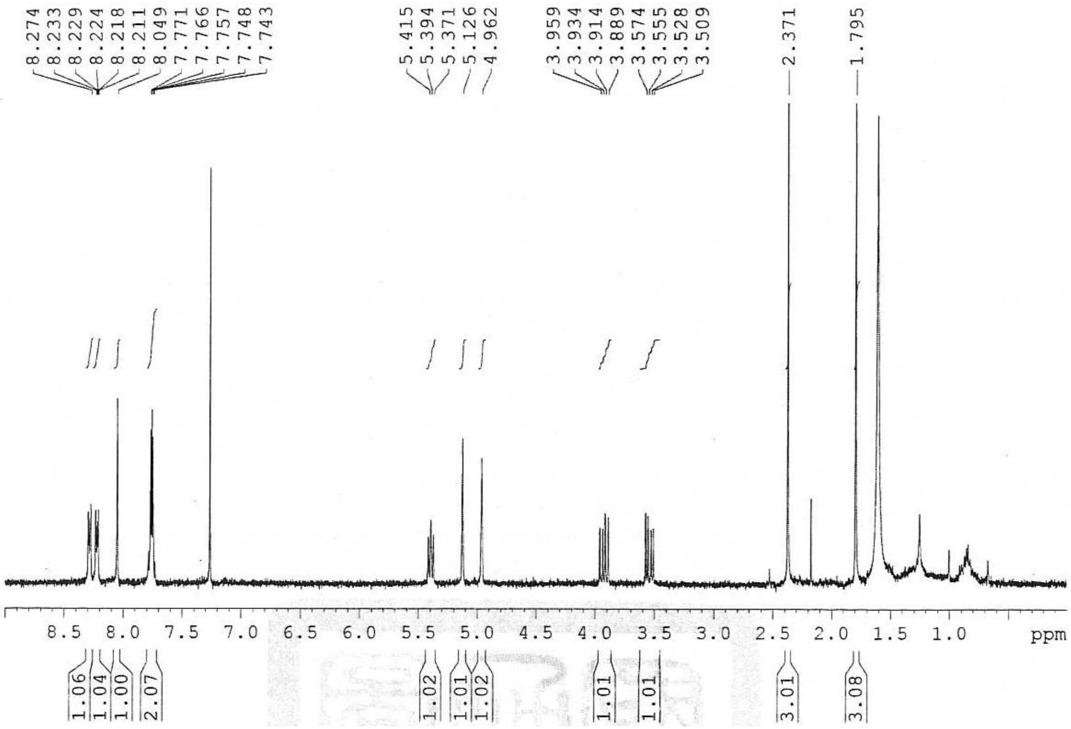

Figure S3. ^{13}C and DEPT NMR spectrum of **1** ($CDCl_3$, 100 MHz).

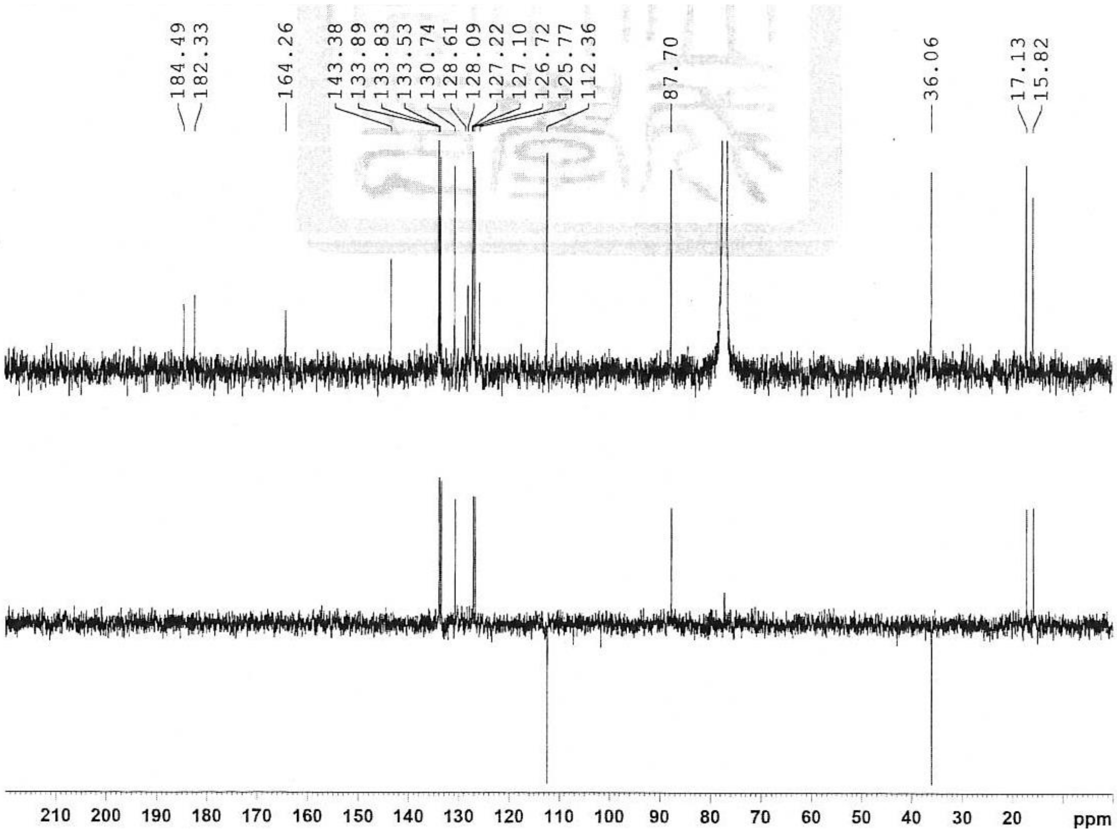

Figure S4. HMBC spectrum of **1** ($CDCl_3$, 400 MHz).

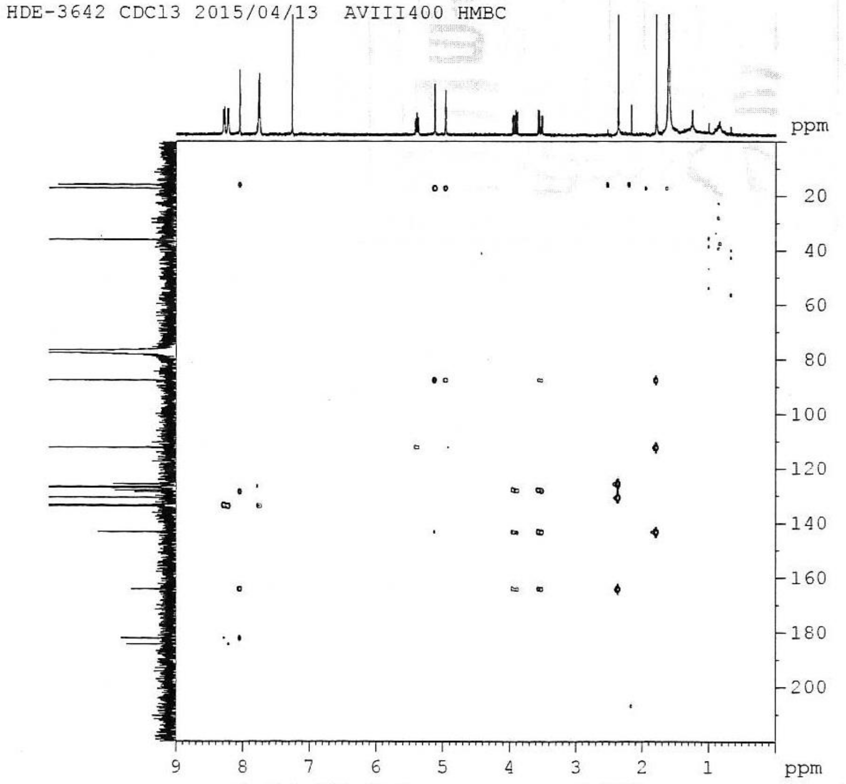

Figure S5. NOESY spectrum of **1** ($CDCl_3$, 400 MHz).

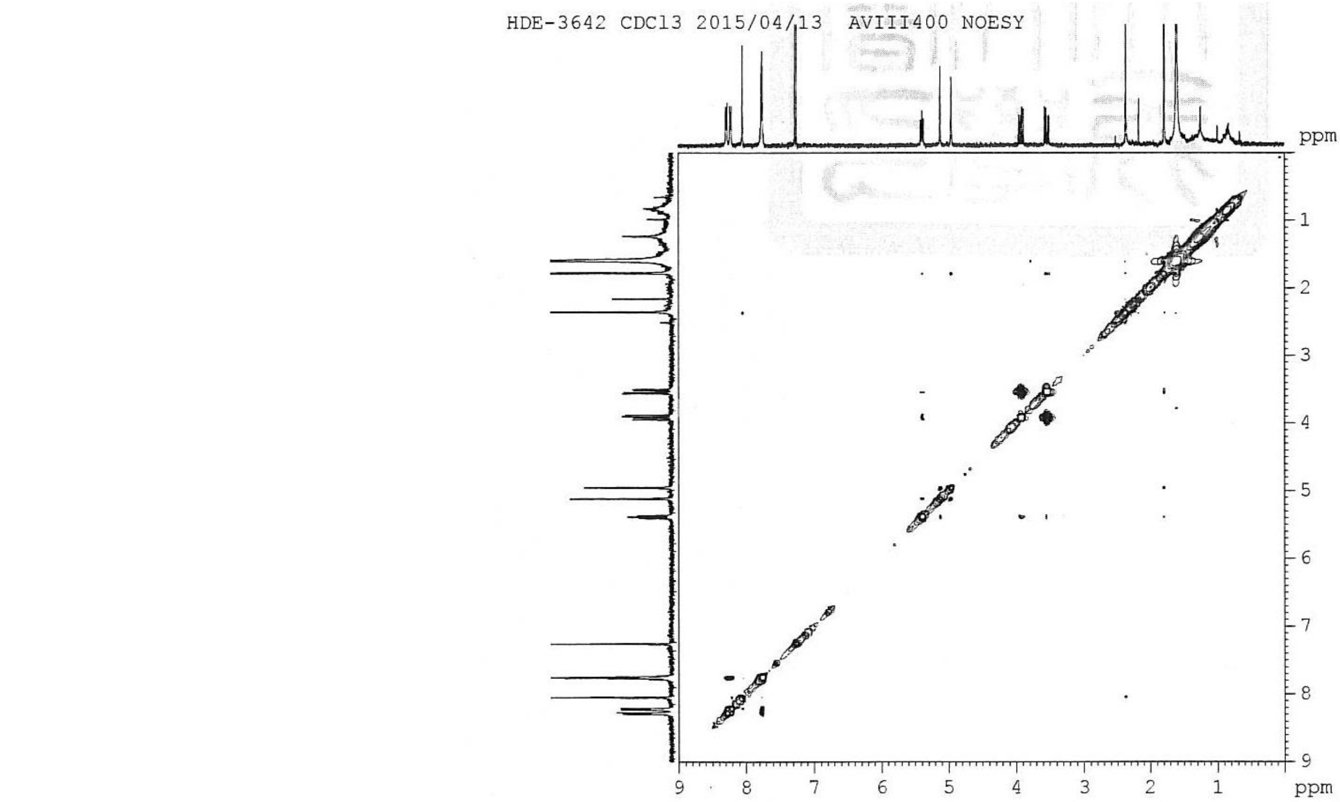

Figure S6. COSY spectrum of **1** ($CDCl_3$, 400 MHz).

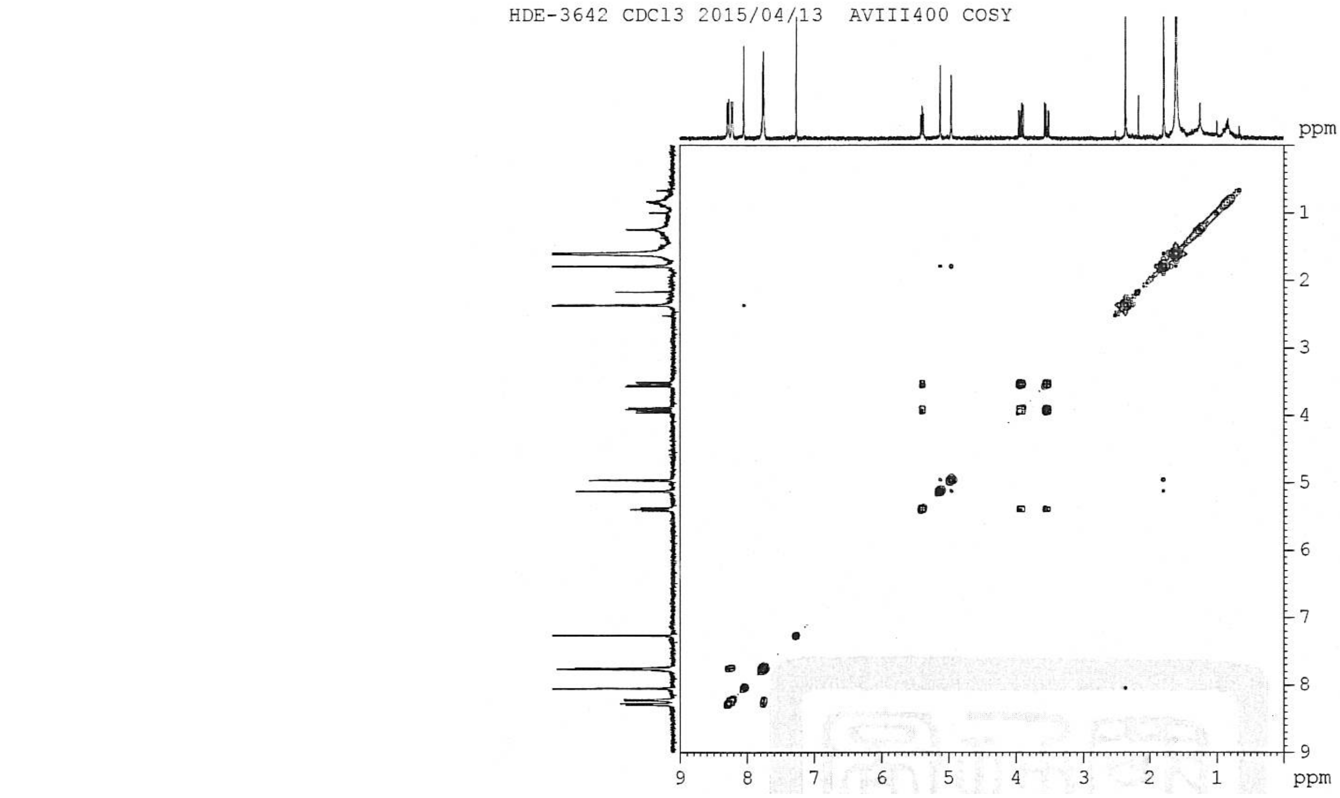

Figure S7. HSQC spectrum of **1** ($CDCl_3$, 400 MHz).

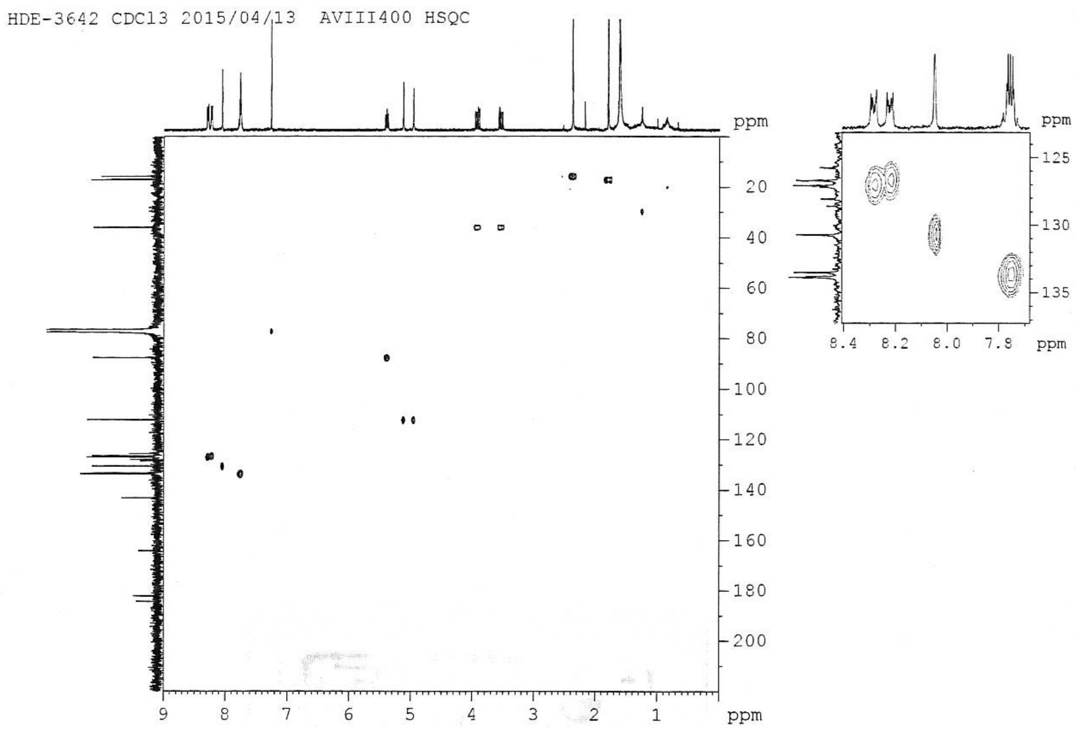

Figure S8. CD spectrum of **1**.

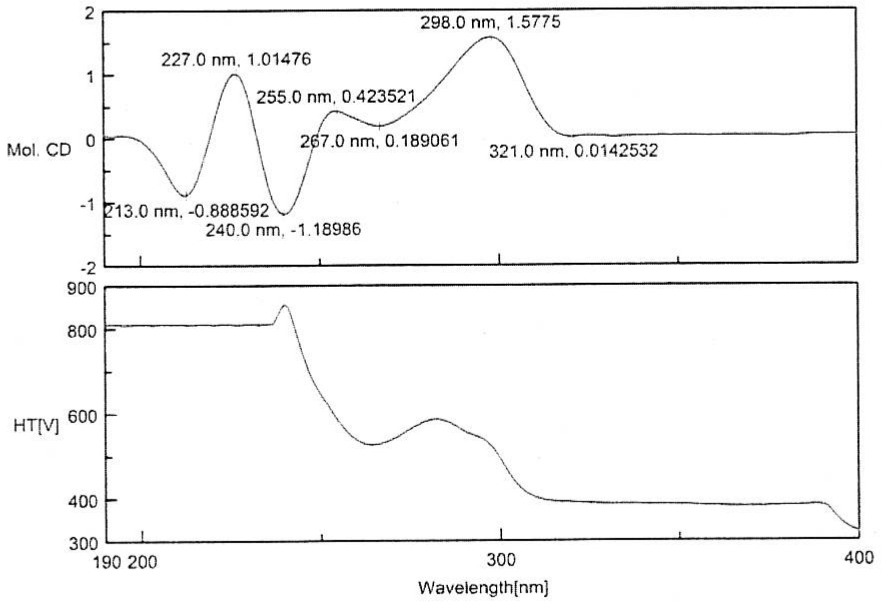

Figure S9. HRMS spectrum of **2**.

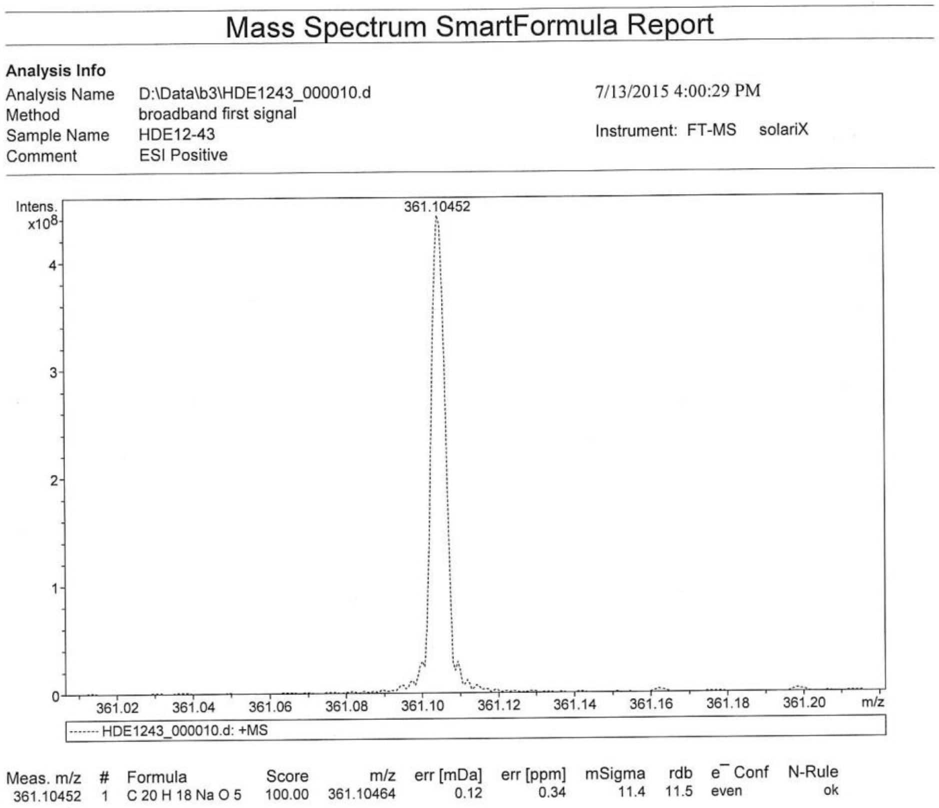

Figure S10. ^{1}H NMR spectrum of **2** (Acetone-d_6, 400 MHz).

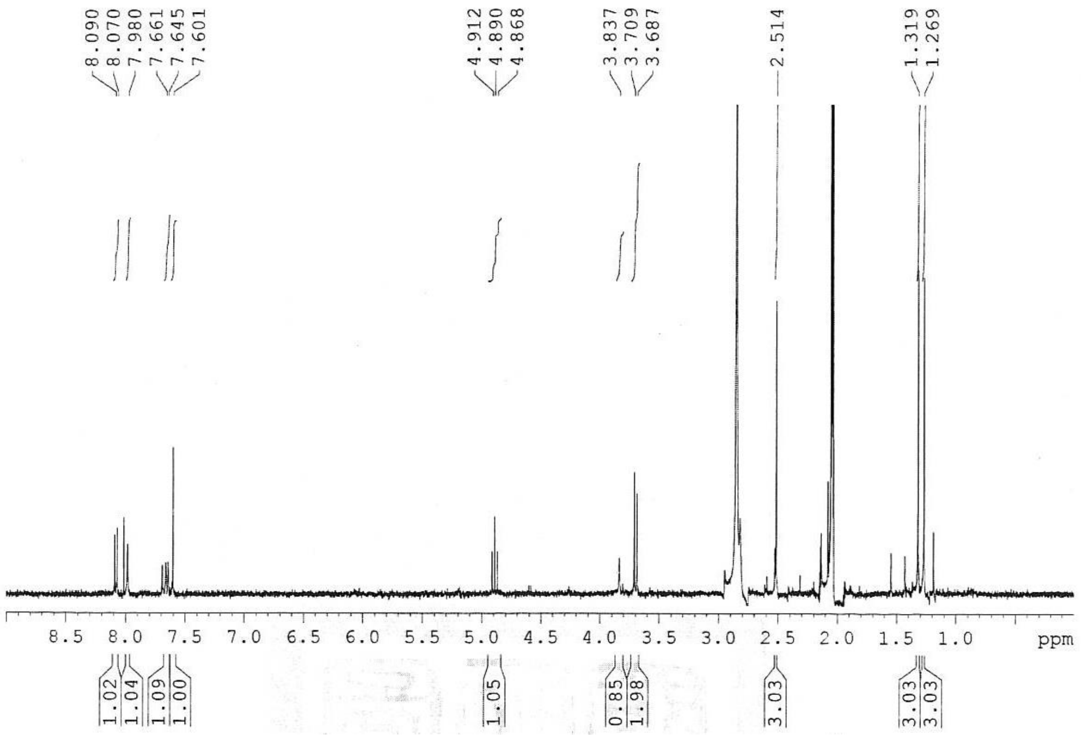

Figure S11. COSY spectrum of **2** (Acetone-d_6, 400 MHz).

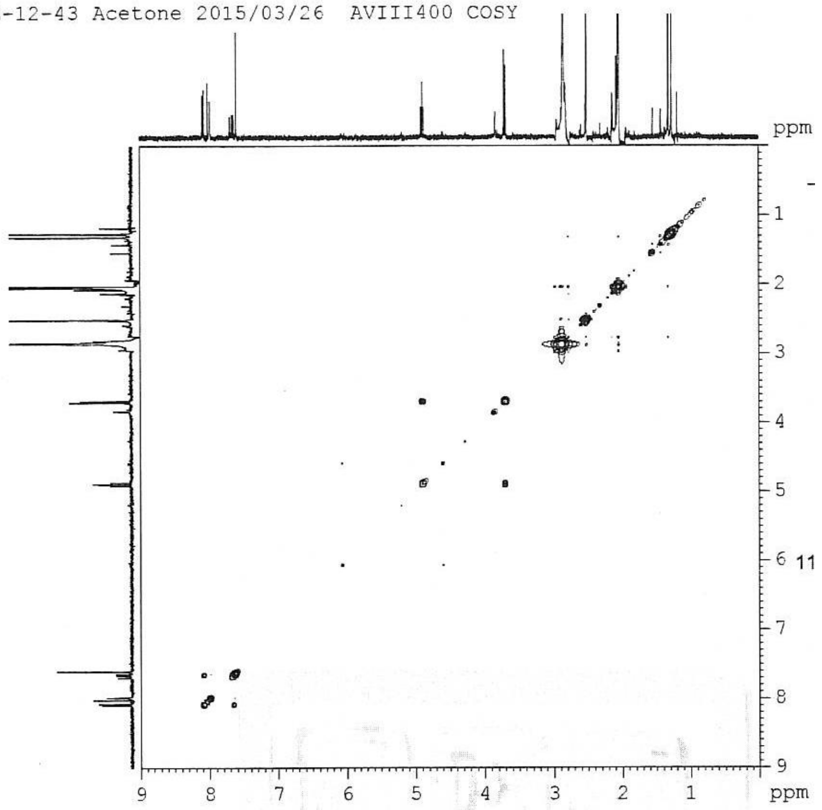

Figure S12. ^{13}C and DEPT NMR spectrum of **2** (Acetone-d_6, 100 MHz).

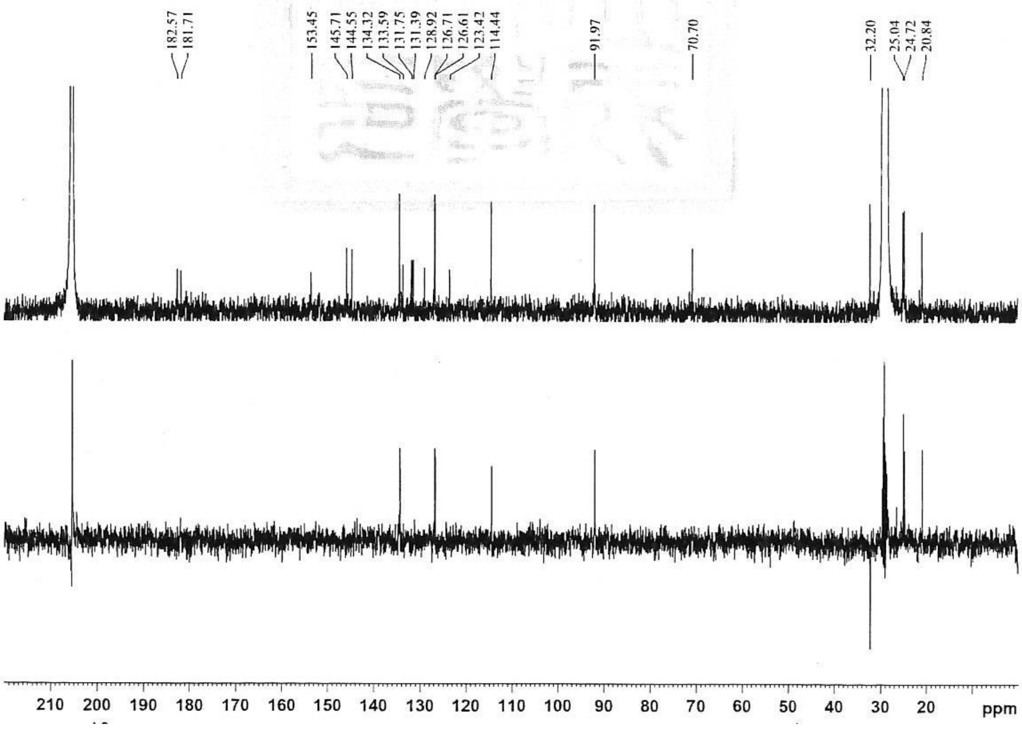

Figure S13. HSQC spectrum of **2** (Acetone-d_6, 400 MHz).

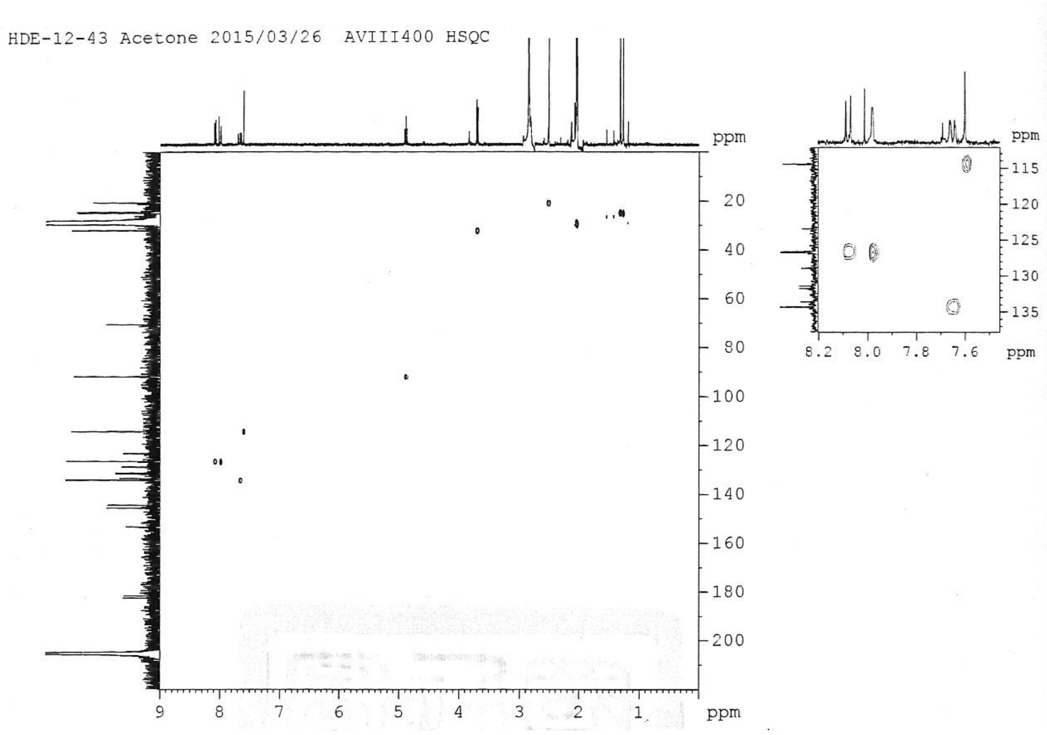

Figure S14. HMBC spectrum of **2** (Acetone-d$_6$, 400 MHz).

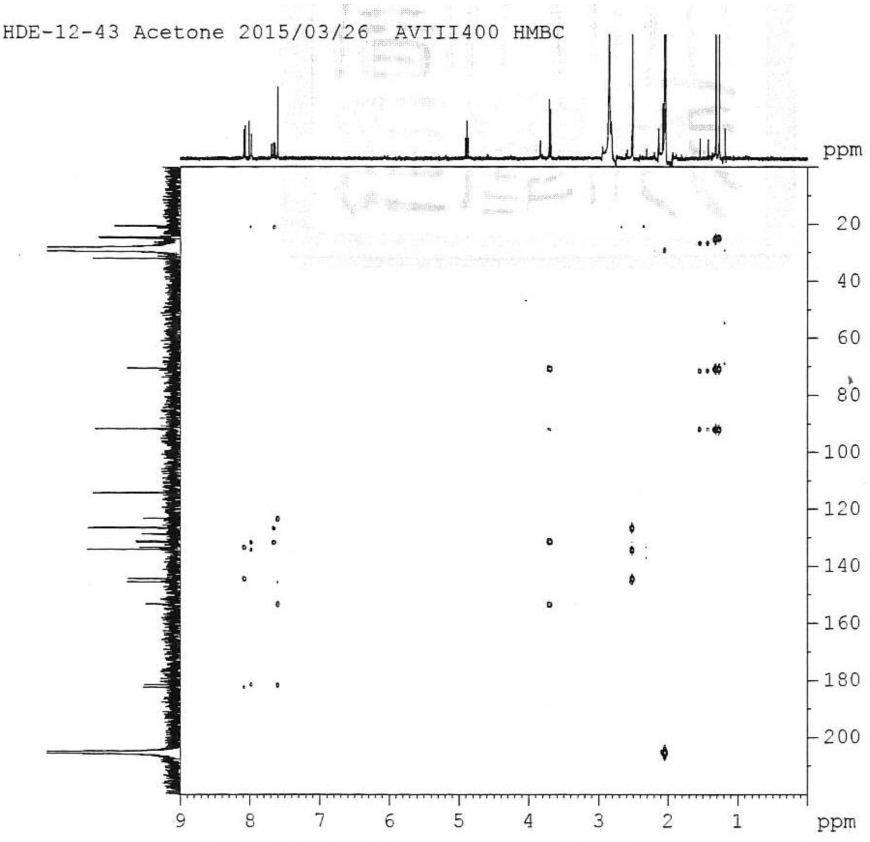

Figure S15. NOESY spectrum of **2** (Acetone-d$_6$, 400 MHz).

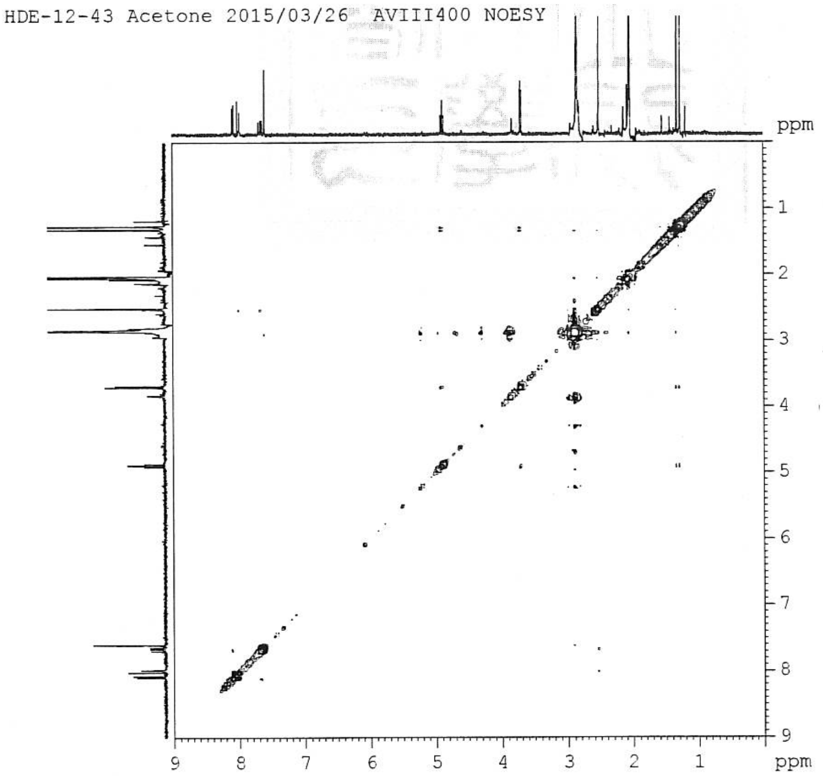

Figure S16. CD spectrum of **2**.

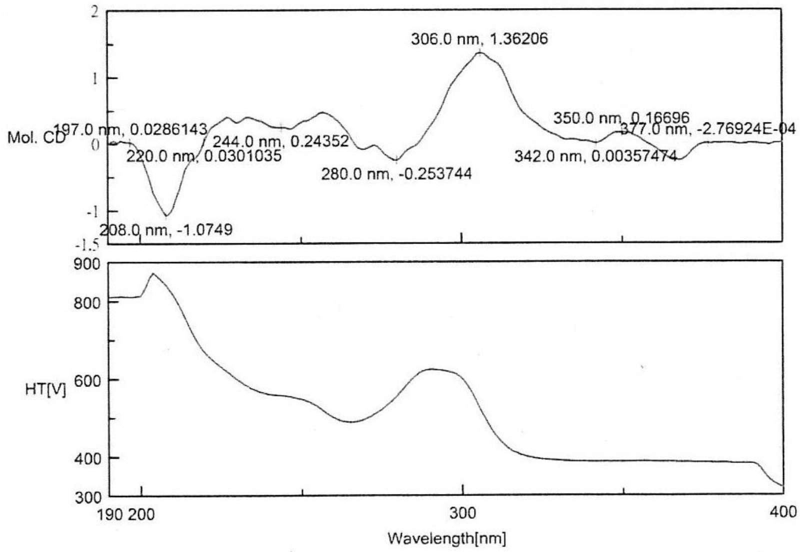

Figure S17. HRMS spectrum of **3**.

Mass Spectrum SmartFormula Report

Analysis Info

Analysis Name	D:\Data\h7\HDE1253_000010.d	10/14/2021 1:31:05 PM
Method	broadband first signal	Operator: YU HSIAO-CHING
Sample Name	HDE12-53	Instrument: BRUKER FT-MS solariX
Comment	ESI Negative	

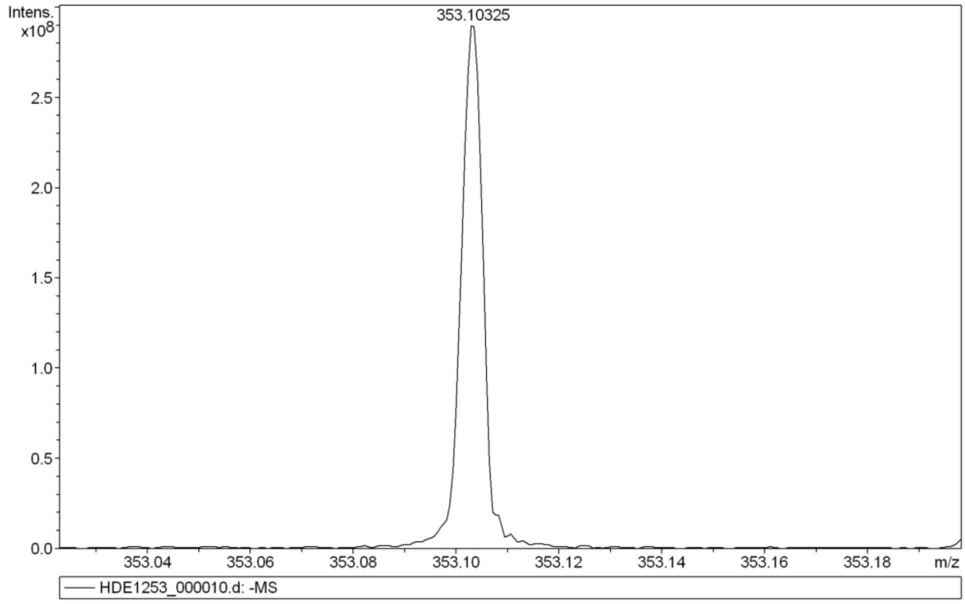

Meas. m/z	#	Formula	Score	m/z	err [mDa]	err [ppm]	mSigma	rdb	e⁻ Conf	N-Rule
353.10325	1	C 20 H 17 O 6	100.00	353.10306	-0.19	-0.54	10.3	12.5	even	ok

Figure S18. ^{1}H NMR spectrum of **3** (CD_3OD, 400 MHz).

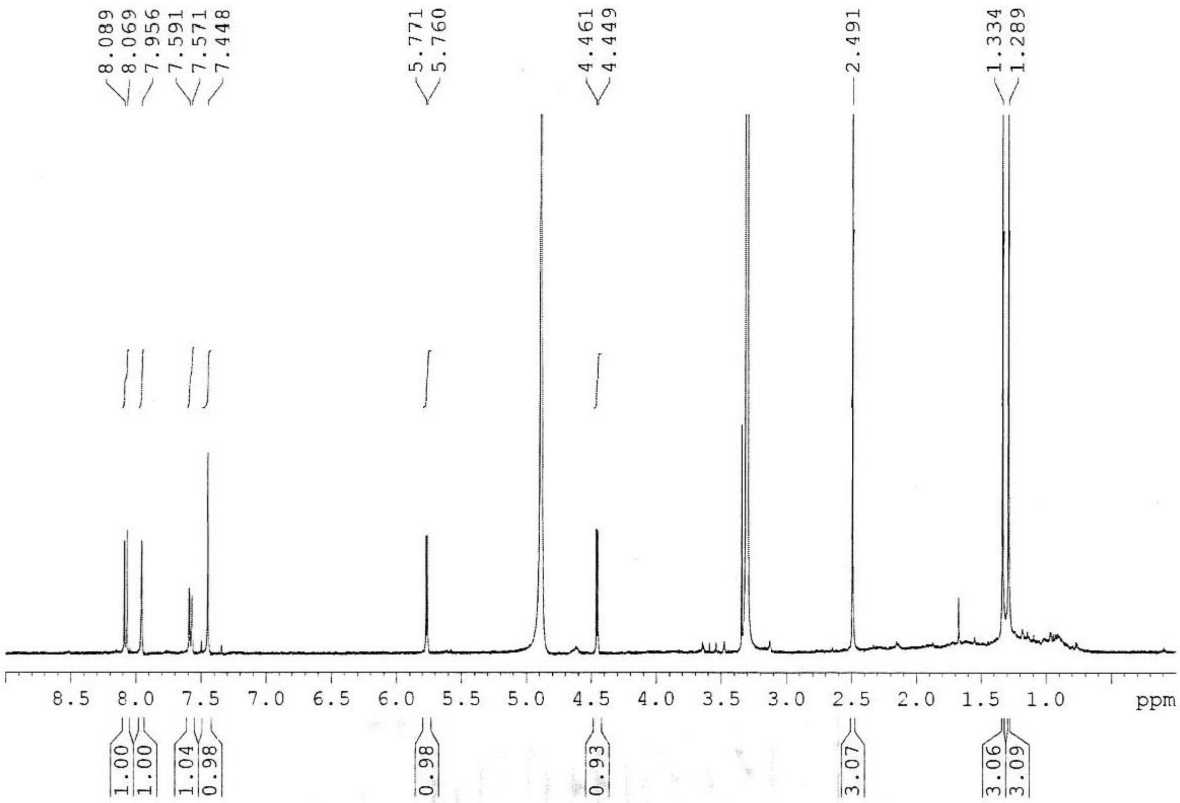

Figure S19. ^{13}C and DEPT NMR spectrum of **3** (CD_3OD, 100 MHz).

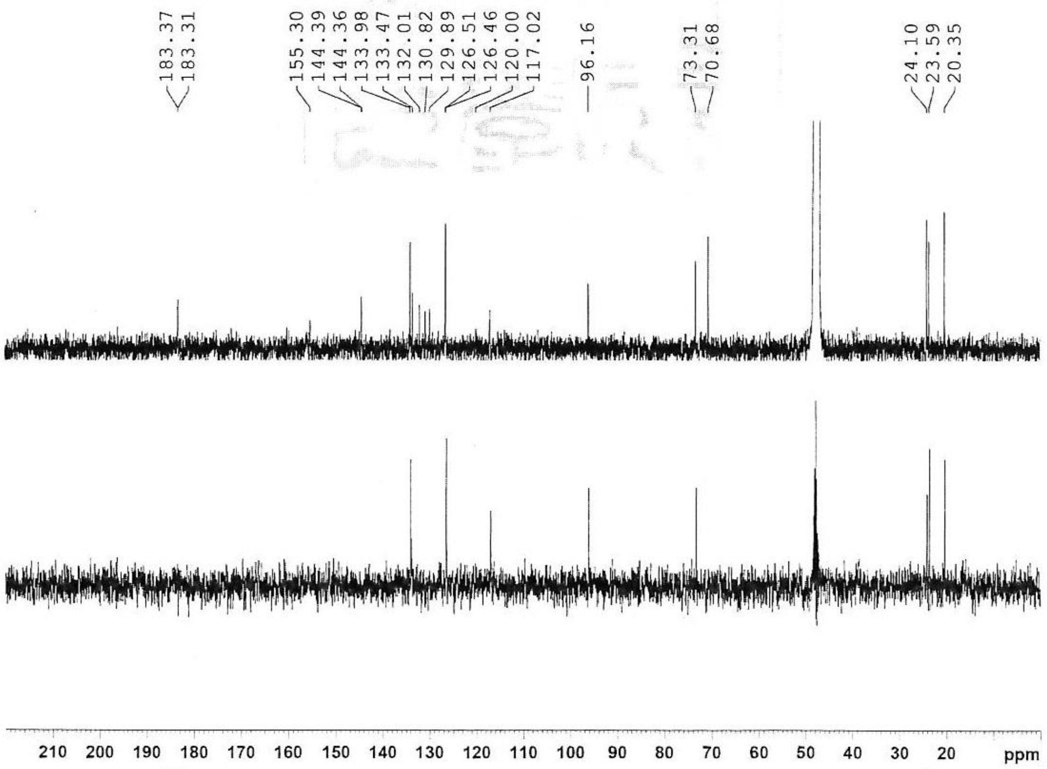

Figure S20. COSY spectrum of **3** (CD_3OD, 400 MHz).

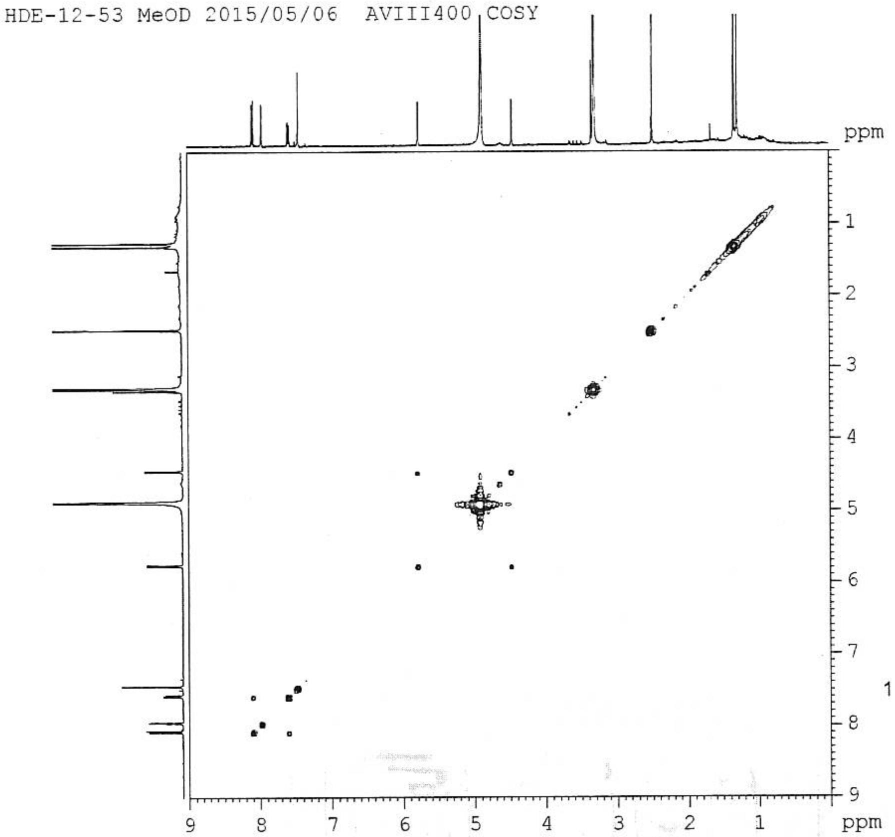

Figure S21. HSQC spectrum of **3** (CD_3OD, 400 MHz).

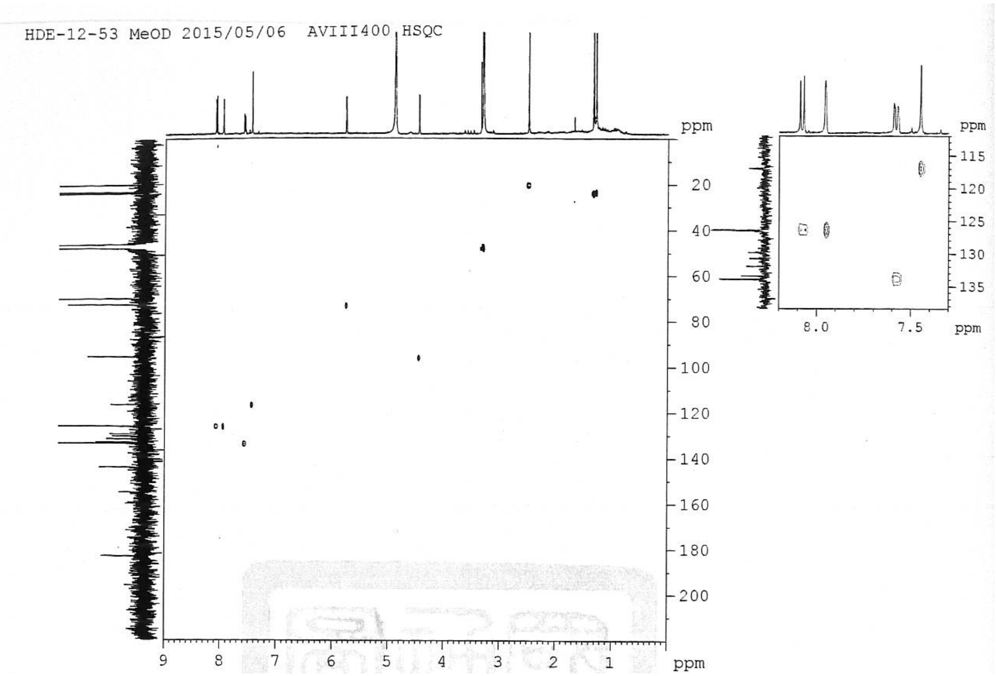

Figure S22. HMBC spectrum of **3** (CD_3OD, 400 MHz).

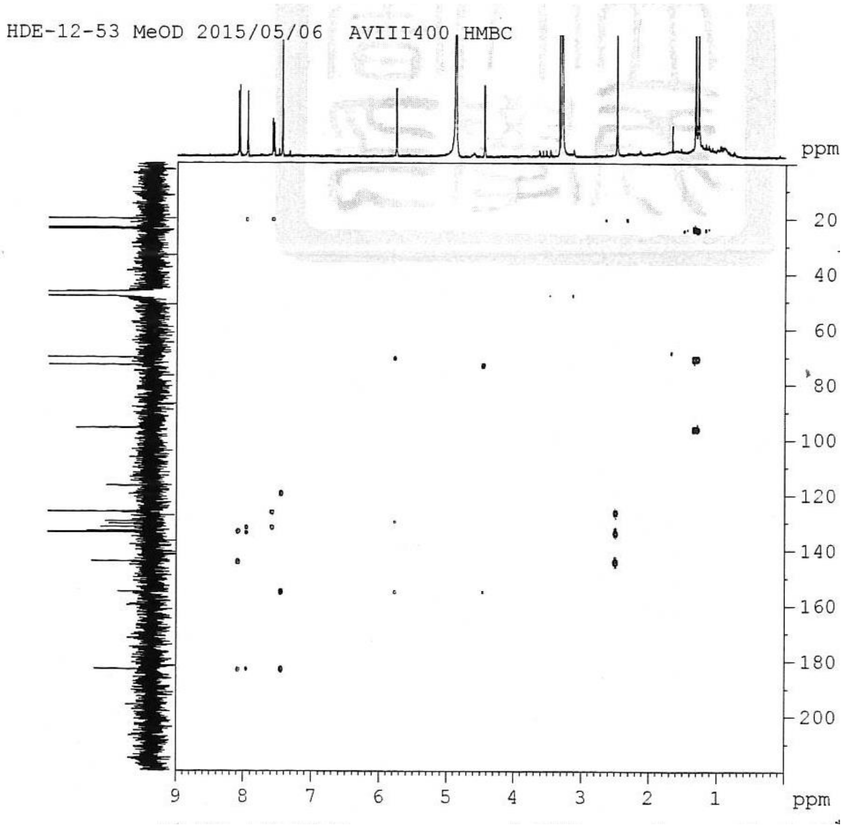

Figure S23. NOESY spectrum of **3** (CD_3OD, 400 MHz).

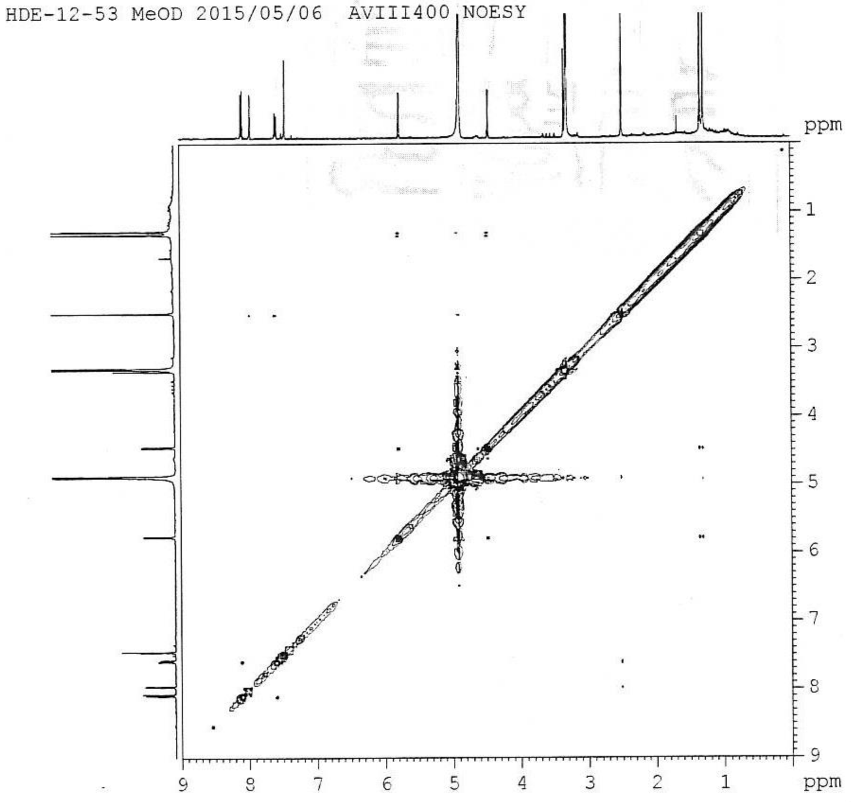

Figure S24. CD spectrum of **3**.

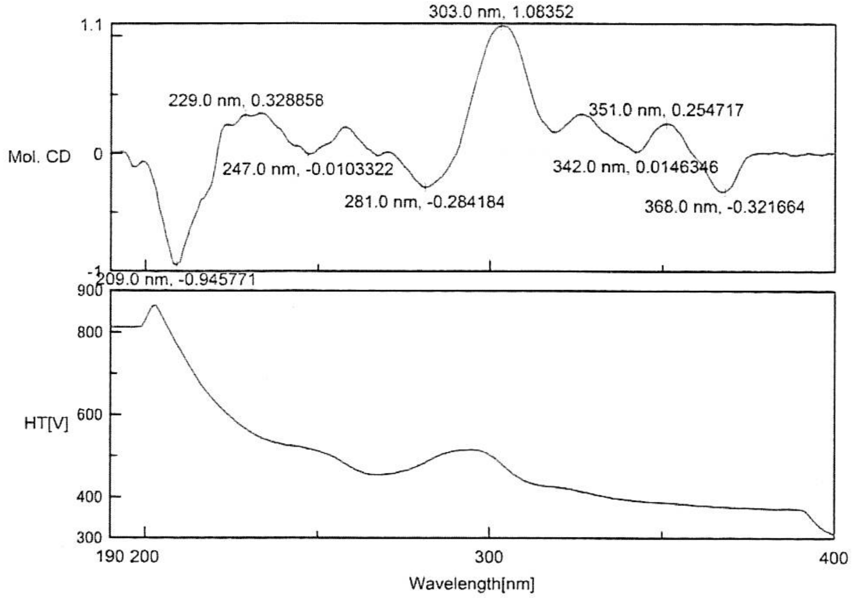

Figure S25. HRMS spectrum of **4**.

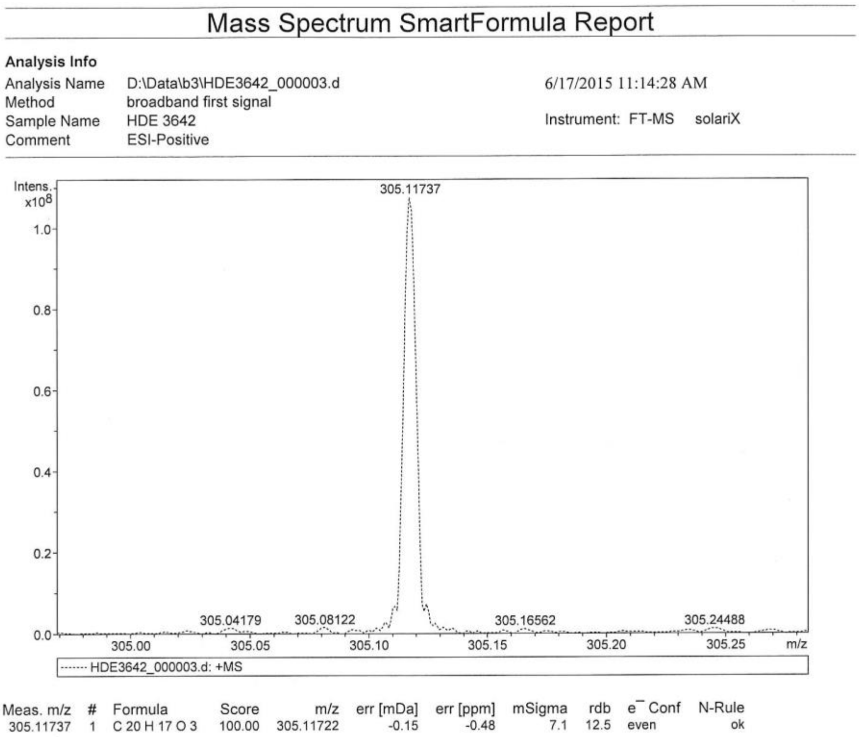

Figure S26. ^{1}H NMR spectrum of **4** ($CDCl_3$, 400 MHz).

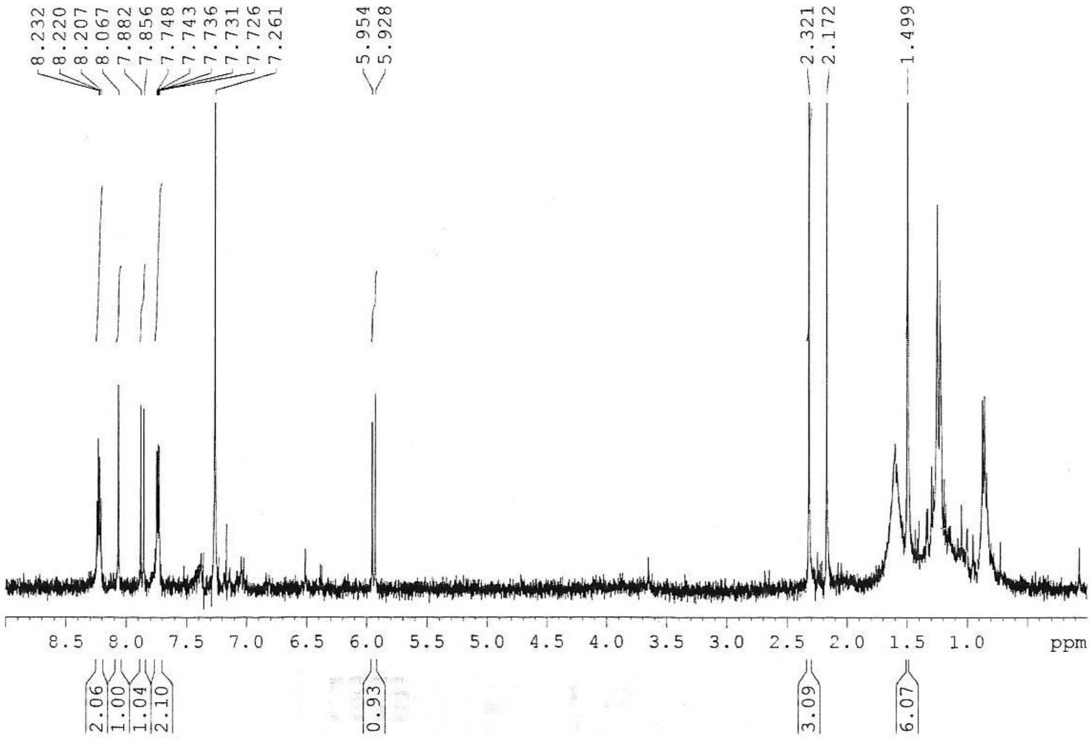

Figure S27. ^{13}C and DEPT NMR spectrum of **4** ($CDCl_3$, 100 MHz).

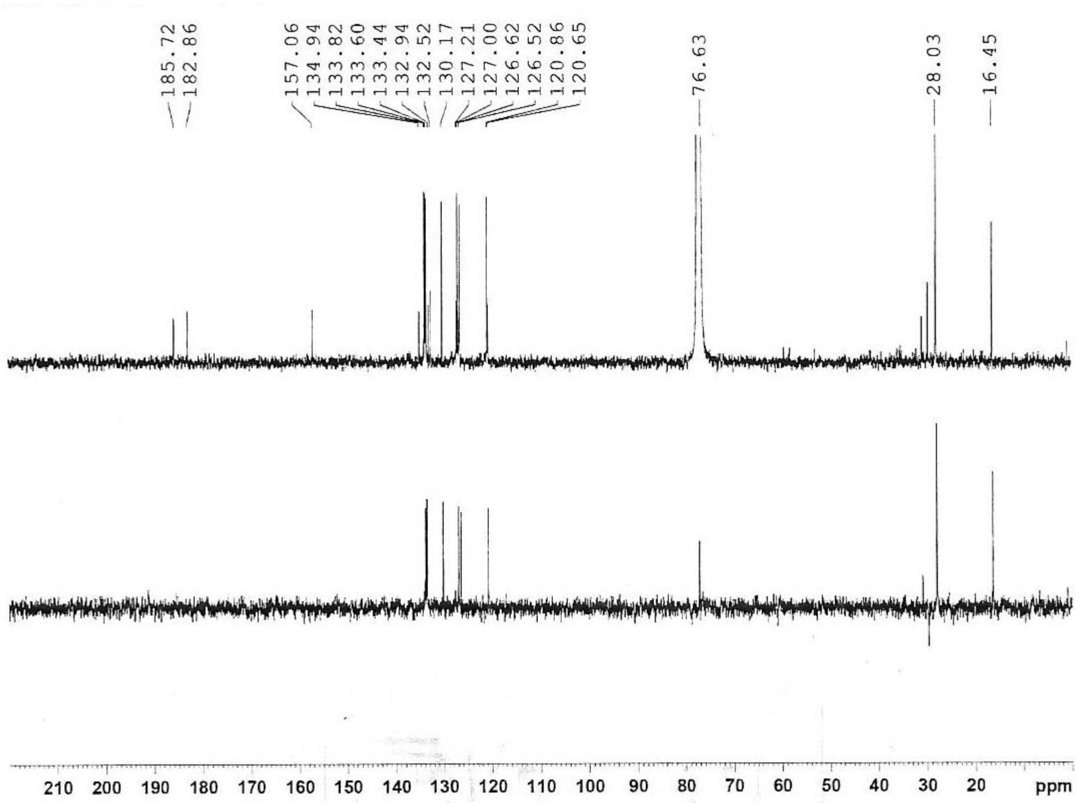

Figure S28. COSY spectrum of **4** ($CDCl_3$, 400 MHz).

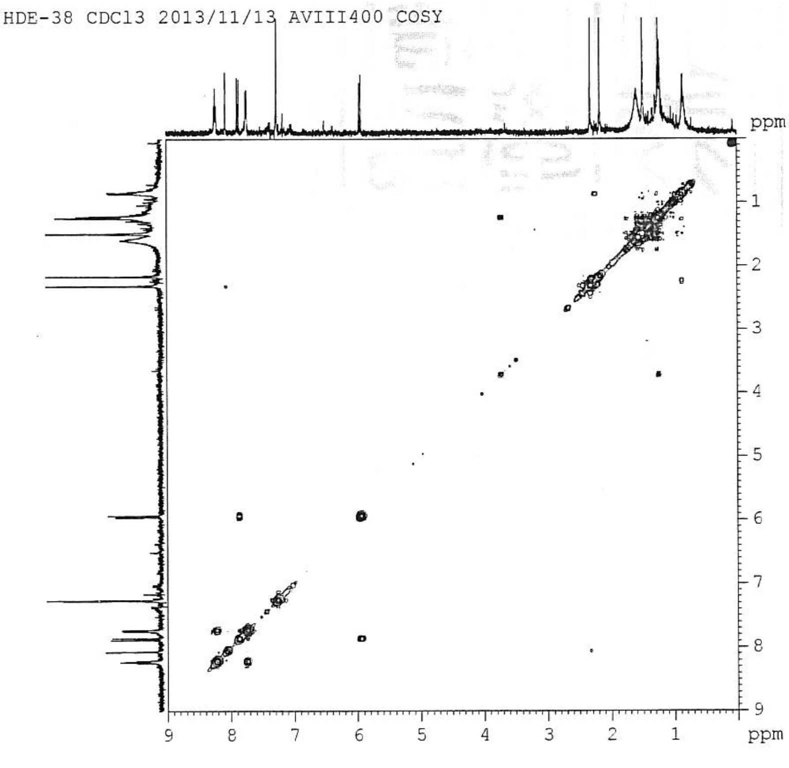

Figure S29. HSQC spectrum of **4** ($CDCl_3$, 400 MHz).

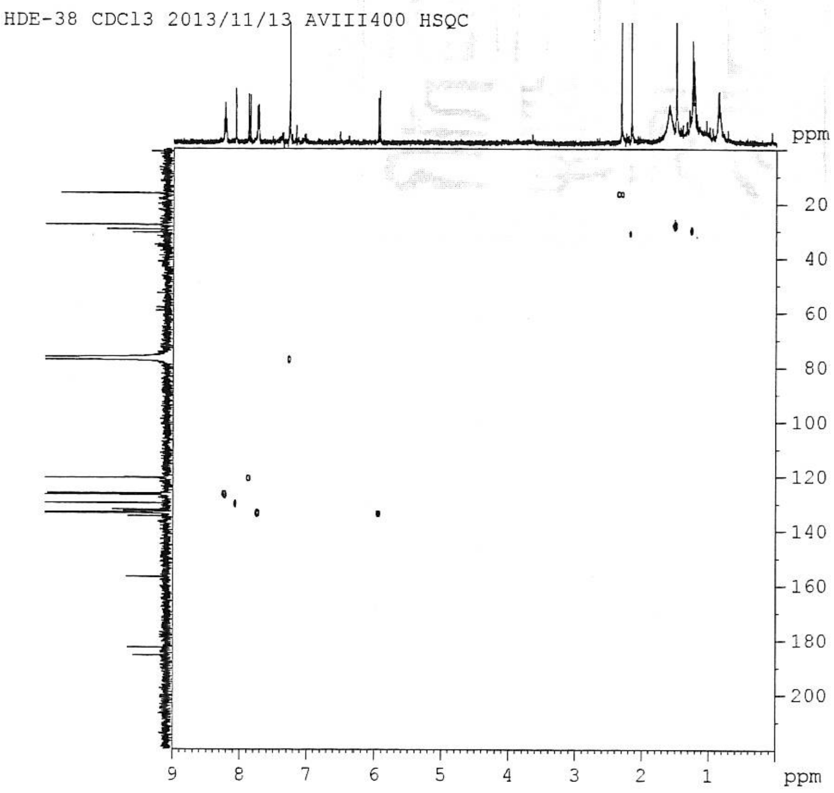

Figure S30. NOESY spectrum of **4** ($CDCl_3$, 400 MHz).

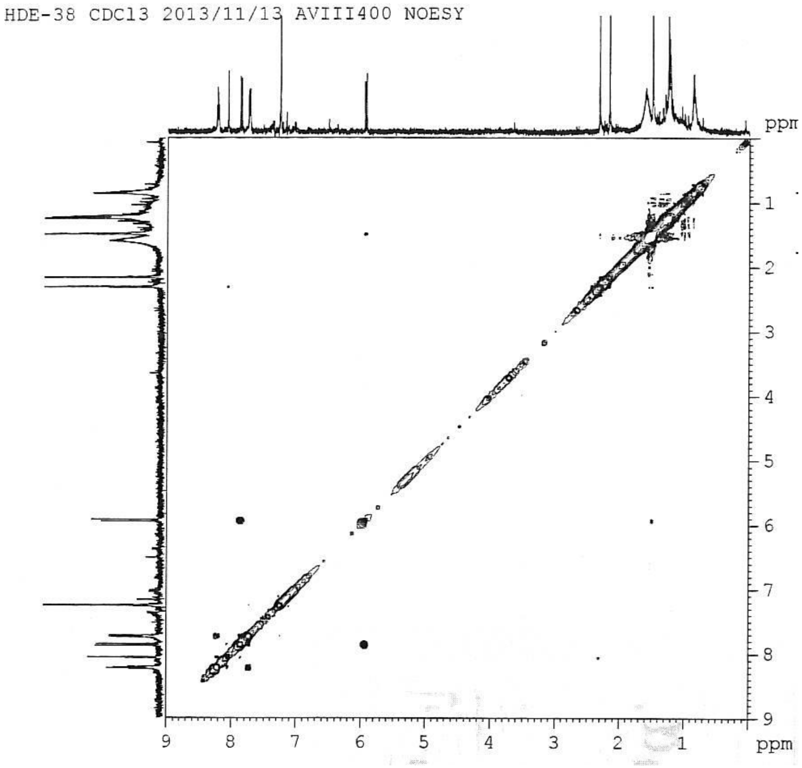

Figure S31. HMBC spectrum of **4** ($CDCl_3$, 400 MHz).

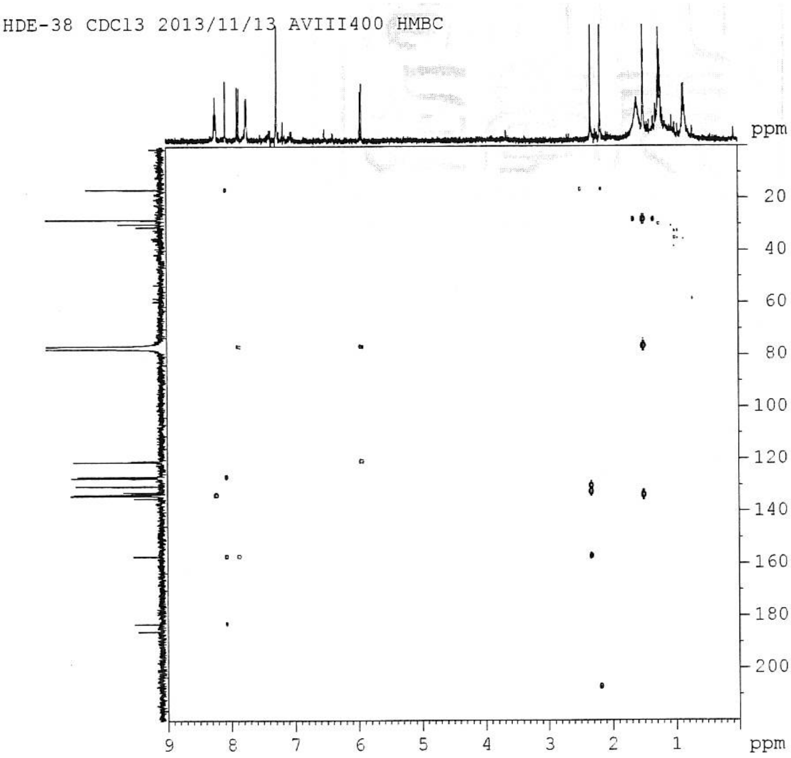

Figure S32. HRMS spectrum of **5**.

Mass Spectrum SmartFormula Report

Analysis Info

Analysis Name D:\Data\b3\HDE6464_000005.d 6/17/2015 11:17:51 AM
Method broadband first signal
Sample Name HDE6464 Instrument: FT-MS solariX
Comment ESI Positive

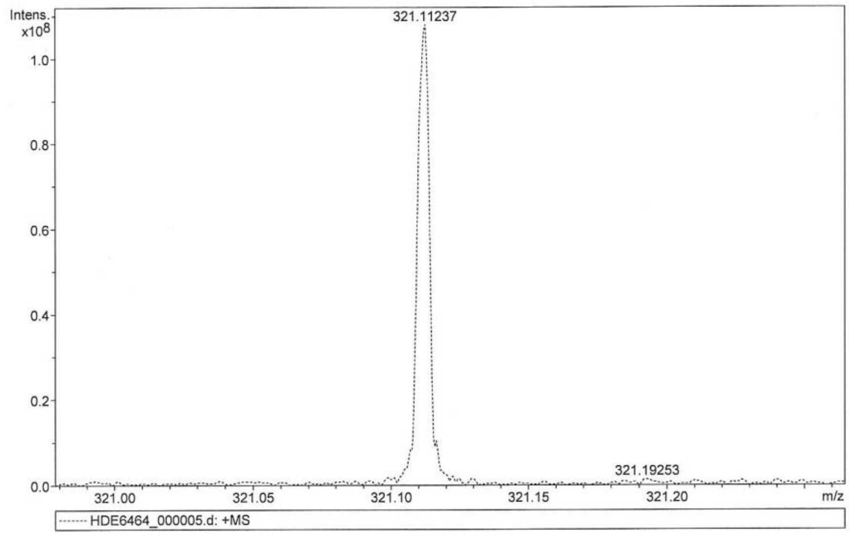

Meas. m/z	#	Formula	Score	m/z	err [mDa]	err [ppm]	mSigma	rdb	e⁻ Conf	N-Rule
321.11237	1	C 20 H 17 O 4	100.00	321.11214	-0.23	-0.73	11.5	12.5	even	ok

Figure S33. ^{1}H NMR spectrum of **5** ($CDCl_3$, 400 MHz).

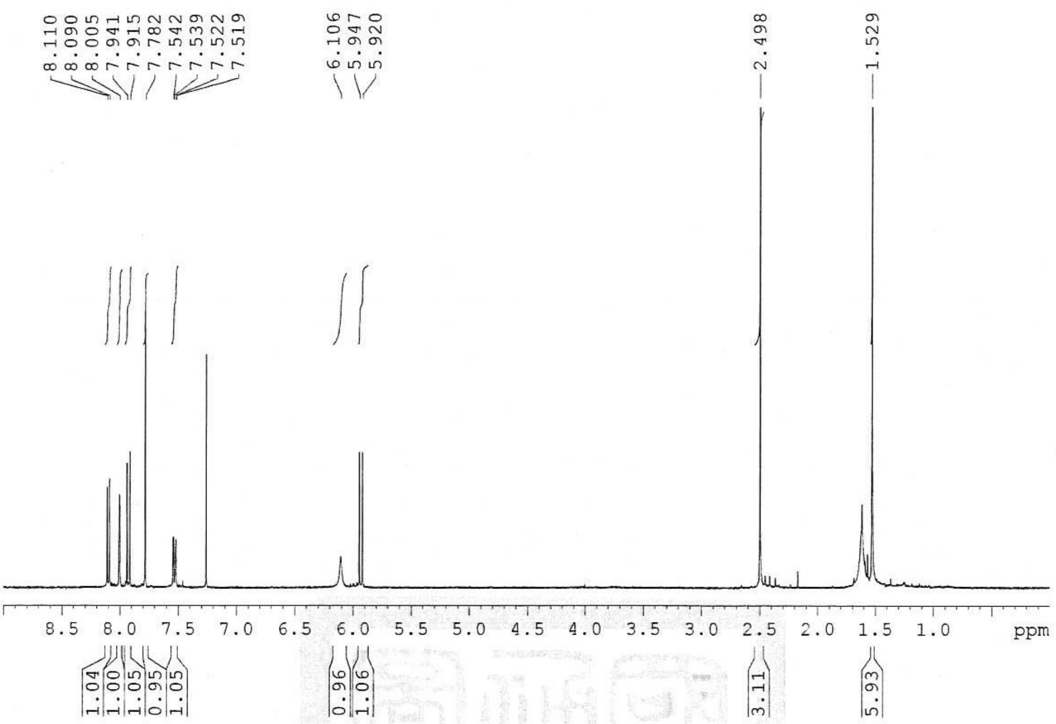

Figure S34. COSY spectrum of **5** ($CDCl_3$, 400 MHz).

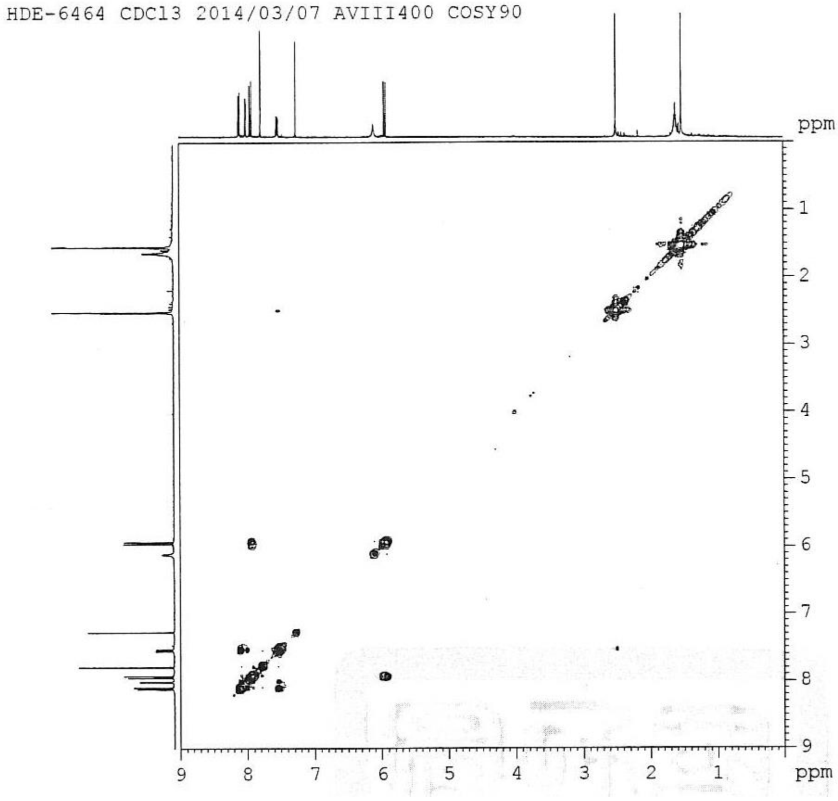

Figure S35. ^{13}C and DEPT NMR spectrum of **5** ($CDCl_3$, 400 MHz).

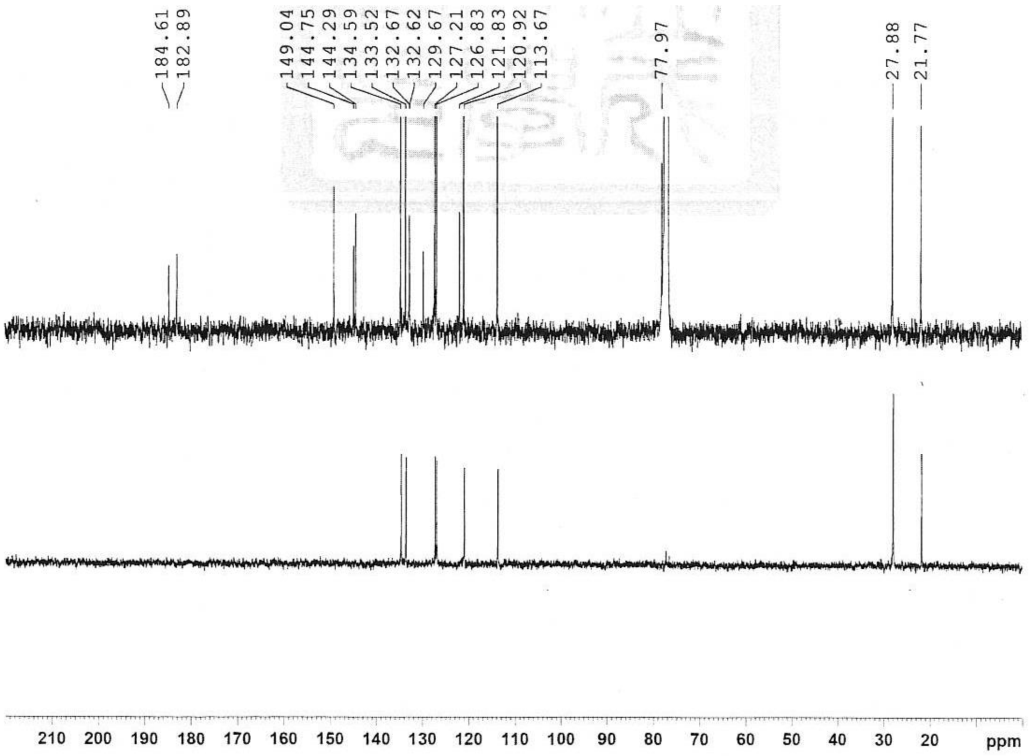

Figure S36. HSQC spectrum of **5** ($CDCl_3$, 400 MHz).

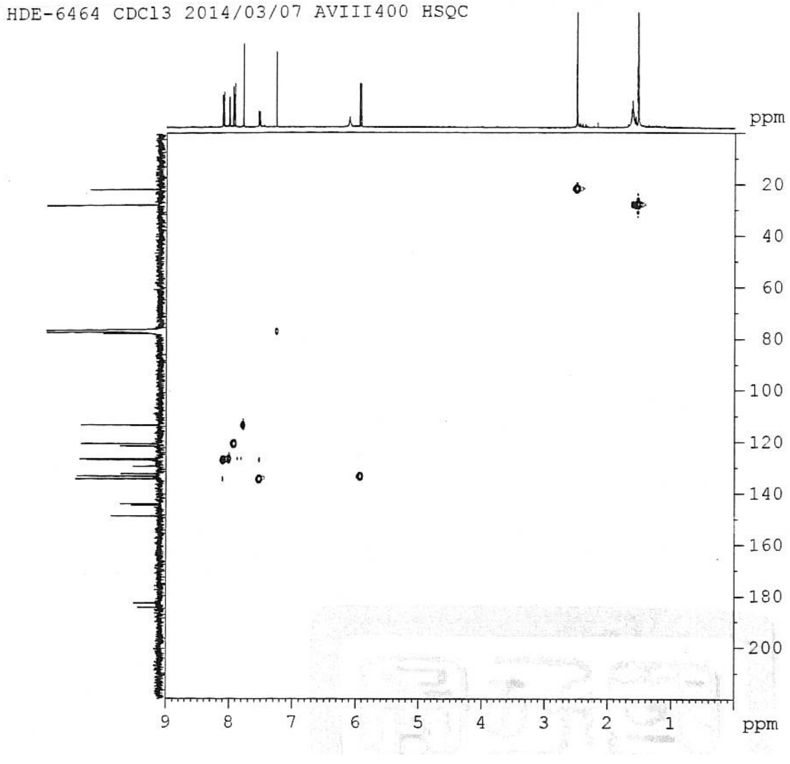

Figure S37. HMBC spectrum of **5** ($CDCl_3$, 400 MHz).

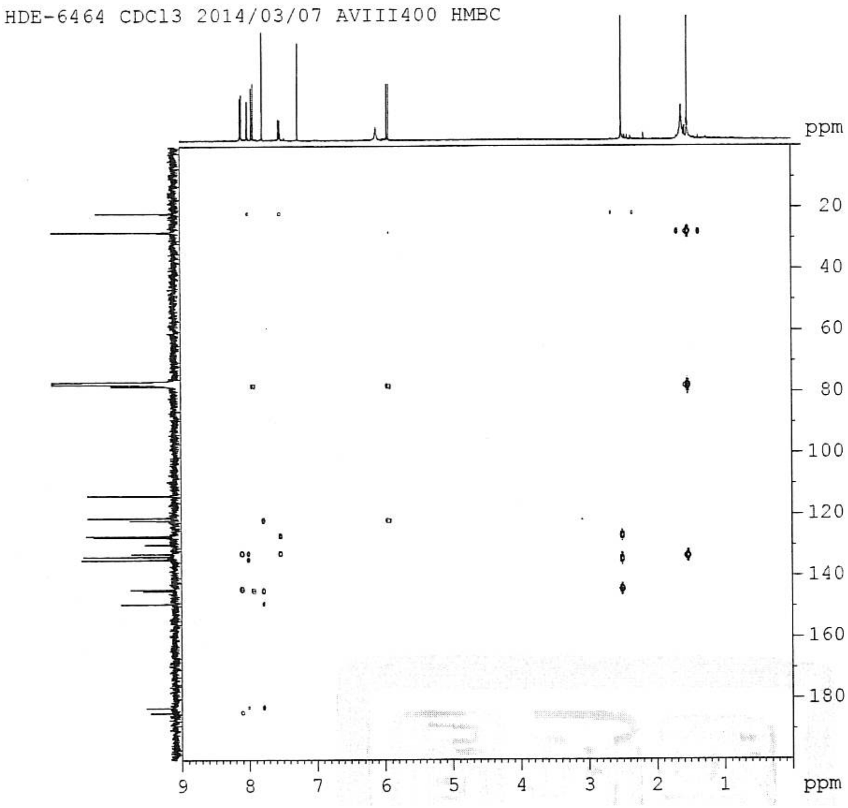

Figure S38. NOESY spectrum of **5** ($CDCl_3$, 400 MHz).

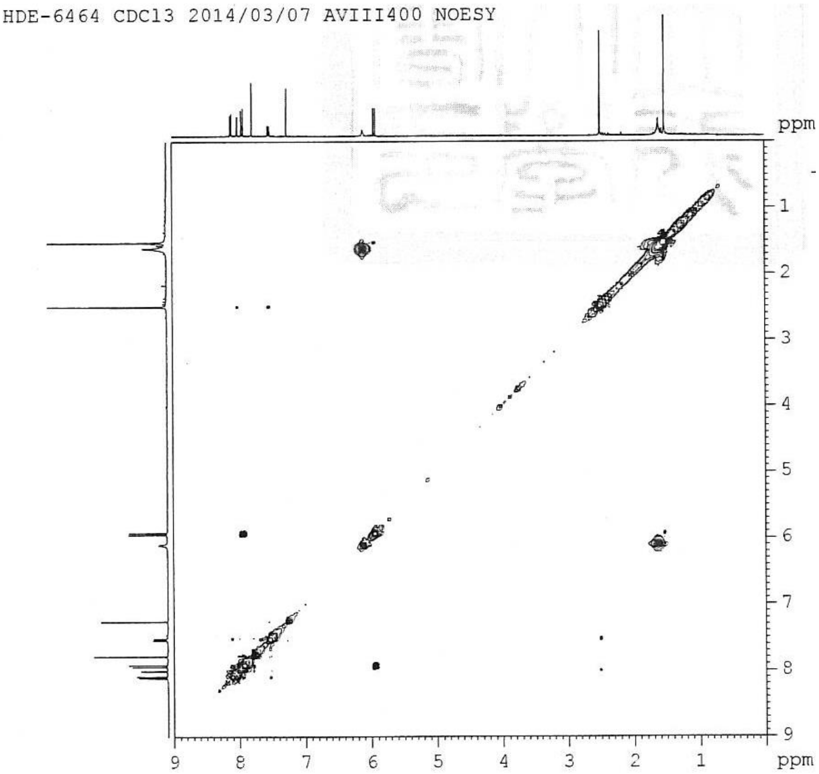

Figure S39. HRMS spectrum of **6**.

Mass Spectrum SmartFormula Report

Analysis Info

Analysis Name	D:\Data\b3\HDE11595_000001.d	4/21/2015 3:02:37 PM
Method	broadband first signal	
Sample Name	HDE11-595	Instrument: FT-MS solariX
Comment	ESI Positive	

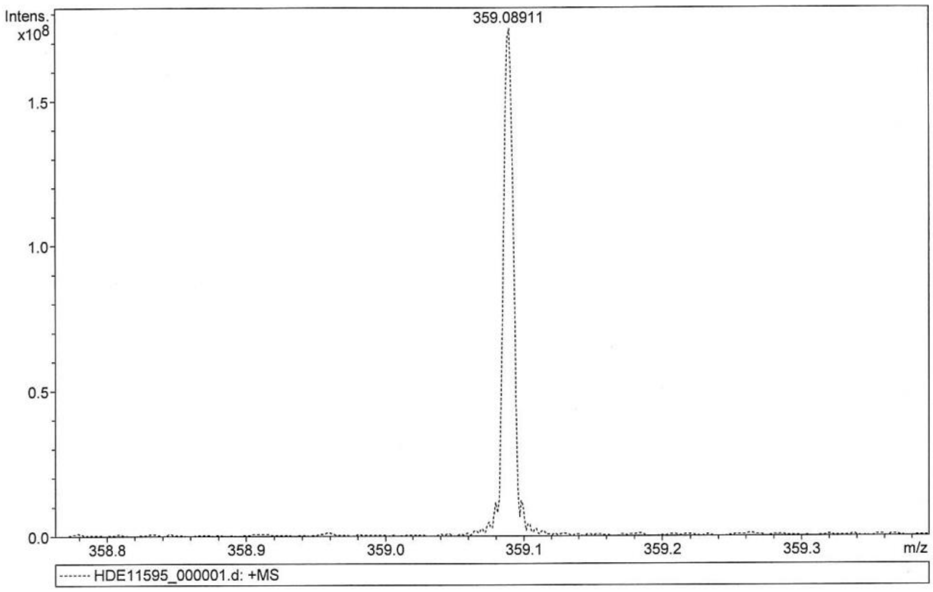

Meas. m/z	#	Formula	Score	m/z	err [mDa]	err [ppm]	mSigma	rdb	e⁻ Conf	N-Rule
359.08911	1	C 20 H 16 Na O 5	100.00	359.08899	-0.11	-0.32	6.9	12.5	even	ok

Figure S40. HRMS spectrum of **7**.

Mass Spectrum SmartFormula Report

Analysis Info

Analysis Name	D:\Data\b3\hde63371_000005.d	4/21/2015 3:17:43 PM
Method	broadband first signal	
Sample Name	HDE63371	Instrument: FT-MS solariX
Comment	ESI-Positive	

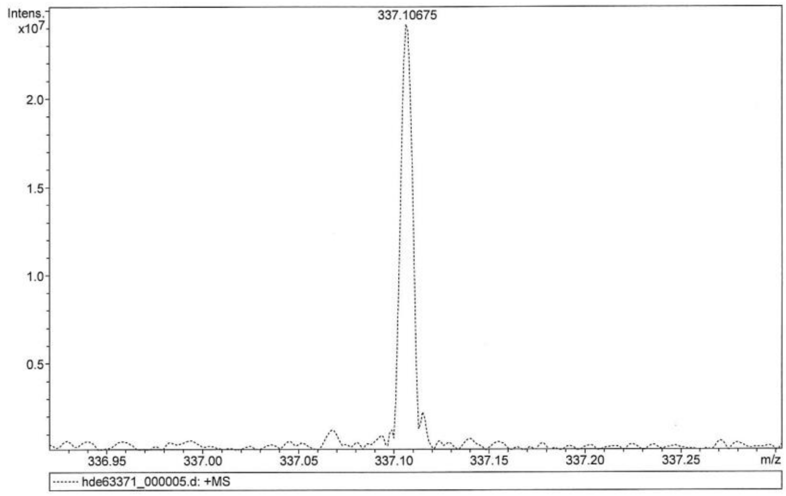

Meas. m/z	#	Formula	Score	m/z	err [mDa]	err [ppm]	mSigma	rdb	e⁻ Conf	N-Rule
337.10675	1	C 20 H 17 O 5	100.00	337.10705	0.30	0.90	18.8	12.5	even	ok

Figure S41. ^{1}H NMR spectrum of **6** (CD_3OD, 700 MHz).

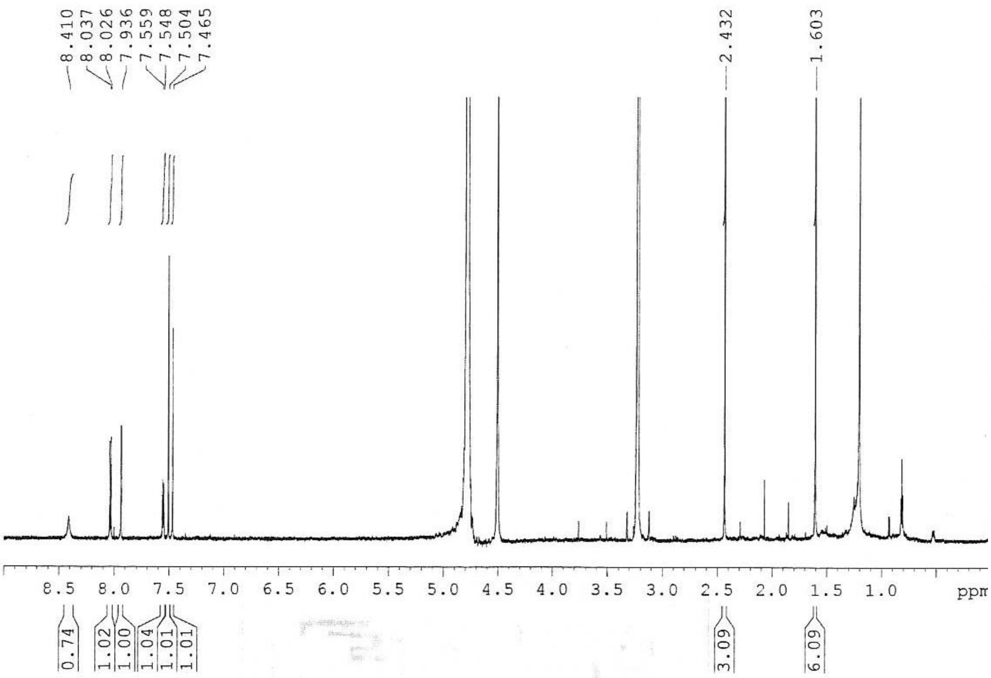

Figure S42. ^{13}C and DEPT NMR spectrum of **6** (CD_3OD, 175 MHz).

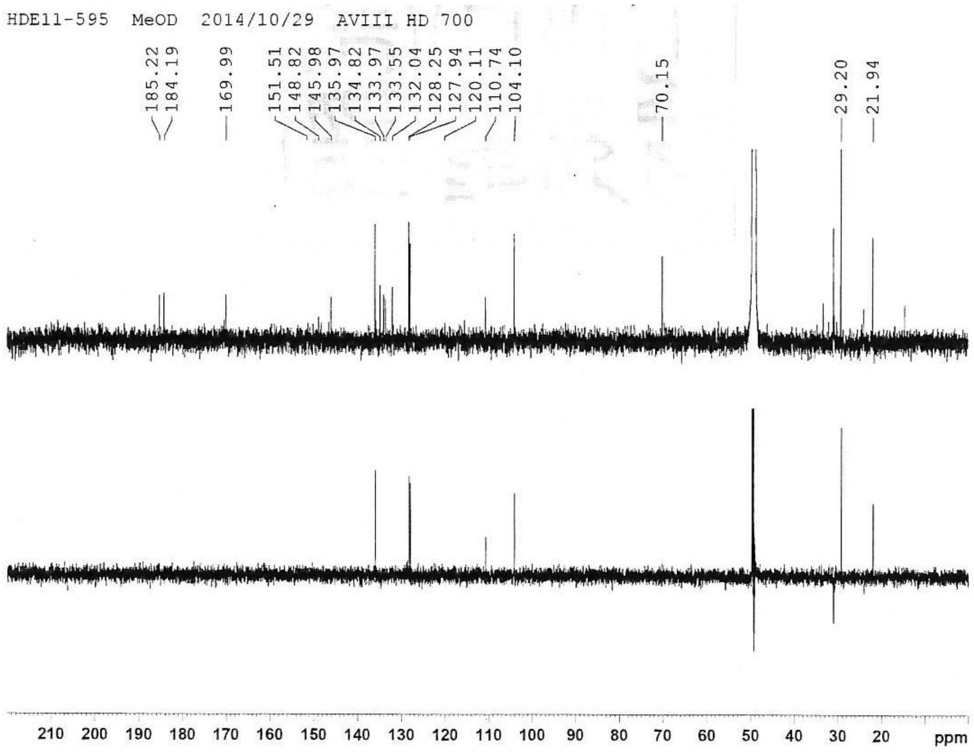

Figure S43. COSY spectrum of **6** (CD_3OD, 700 MHz).

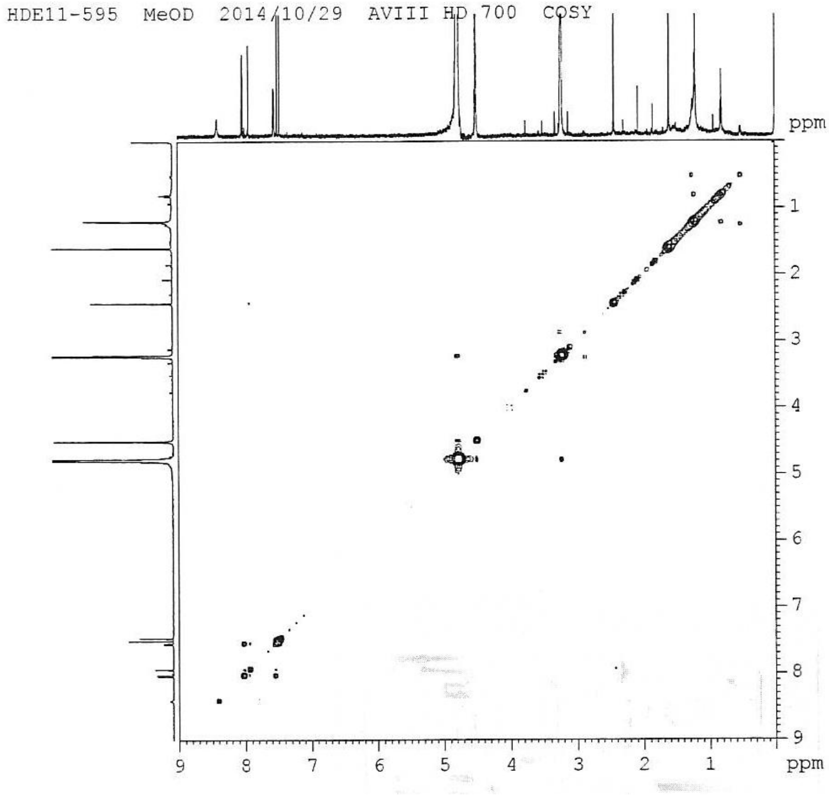

Figure S44. HSQC spectrum of **6** (CD_3OD, 700 MHz).

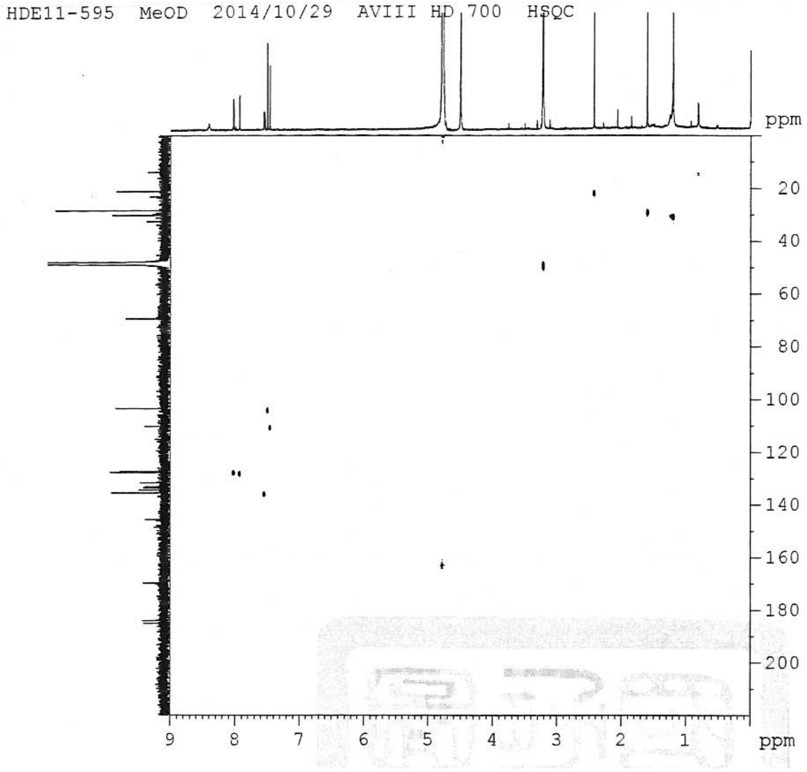

Figure S45. NOESY spectrum of **6** (CD_3OD, 700 MHz).

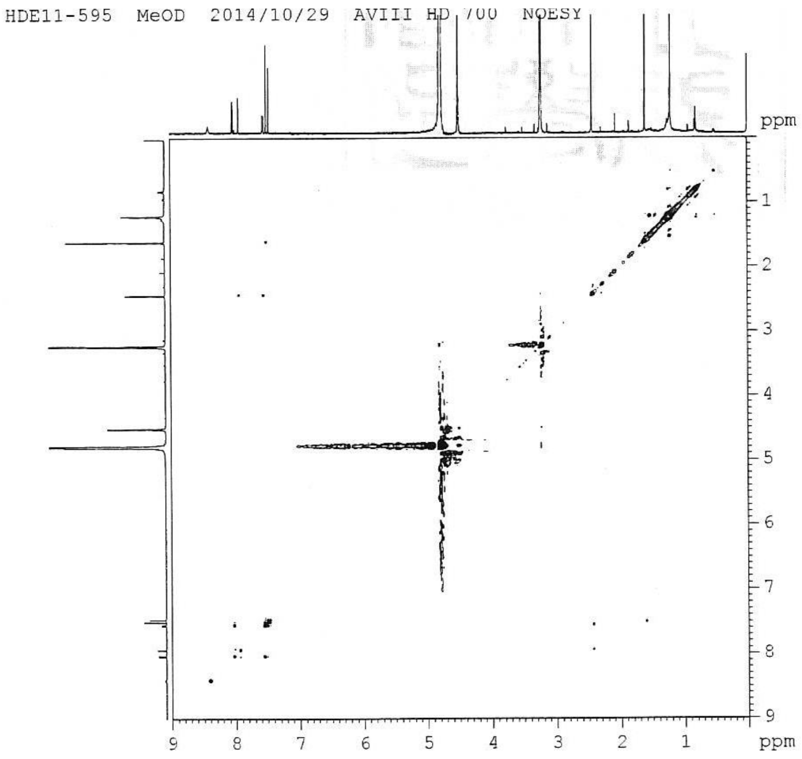

Figure S46. HMBC spectrum of **6** (CD_3OD, 700 MHz).

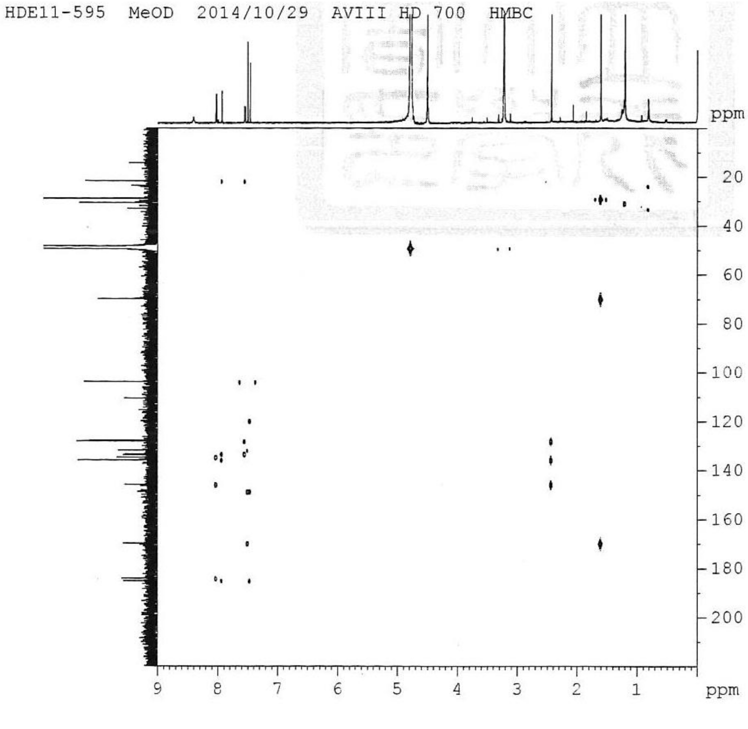

Figure S47. ^{1}H NMR spectrum of **7** ($CDCl_3$, 400 MHz).

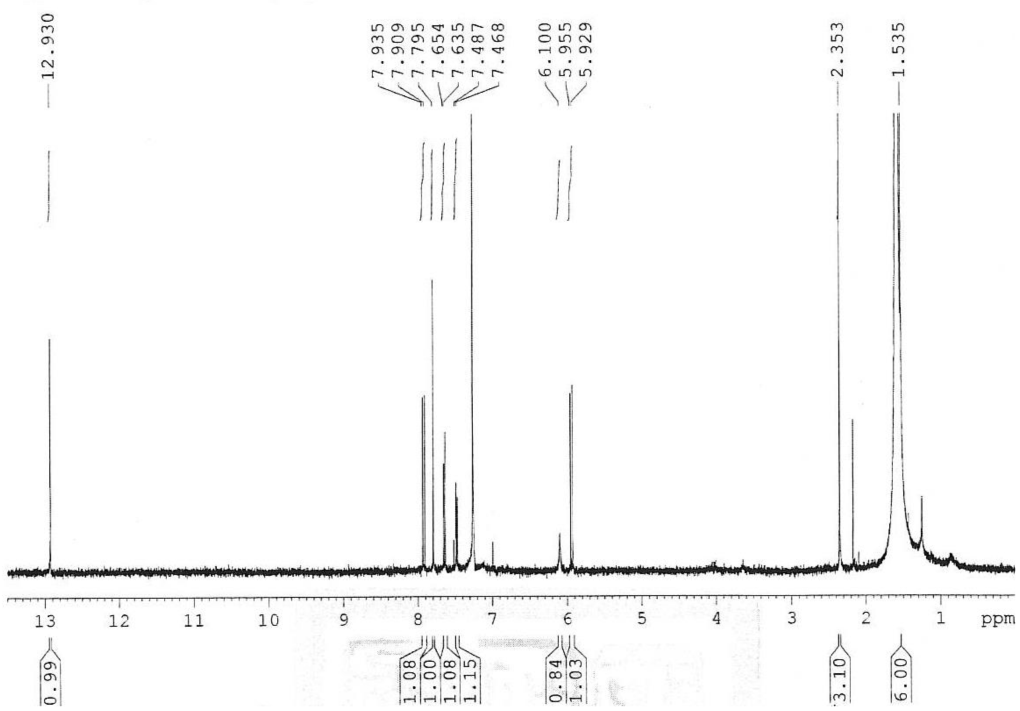

Figure S48. ^{13}C and DEPT NMR spectrum of **7** ($CDCl_3$, 400 MHz).

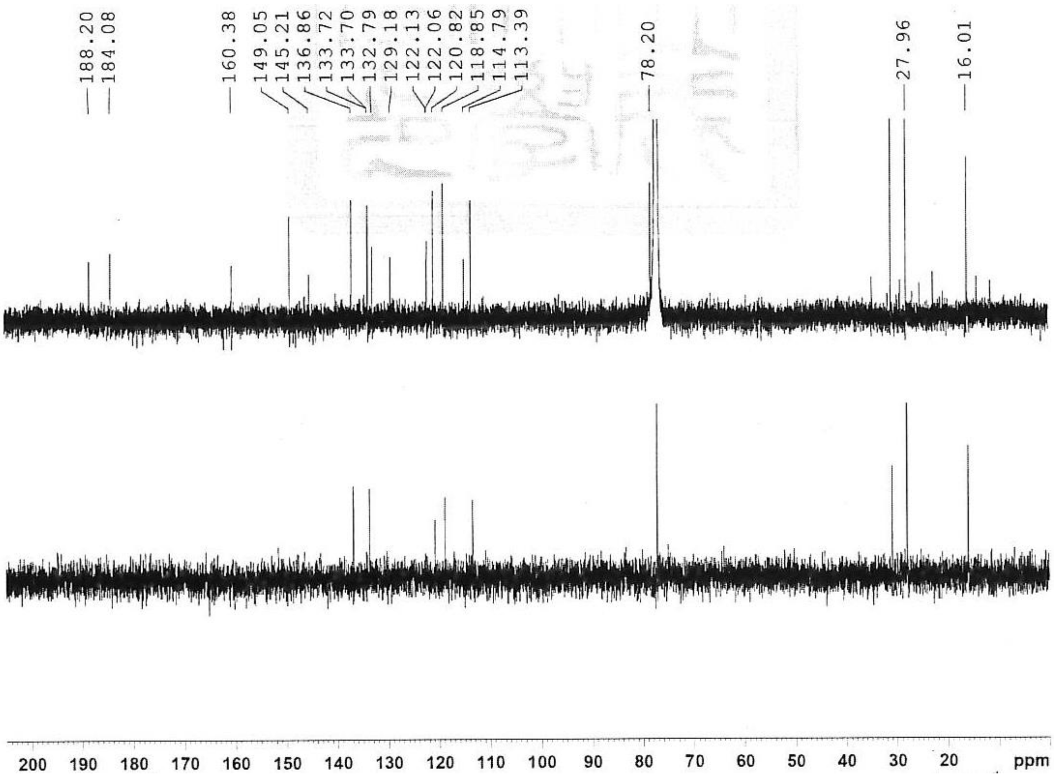

Figure S49. COSY spectrum of **7** ($CDCl_3$, 400 MHz).

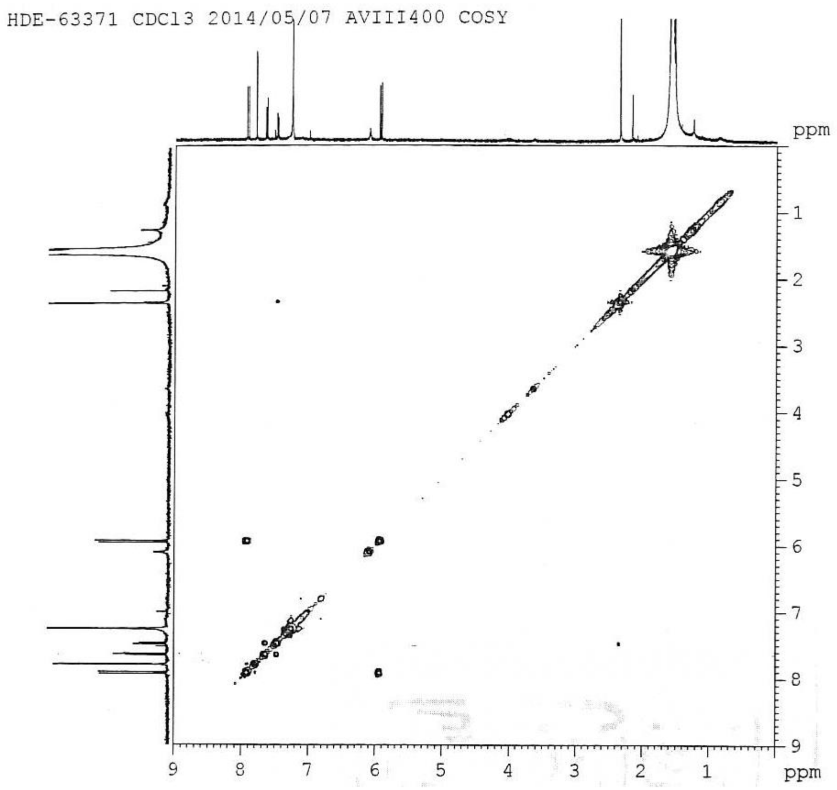

Figure S50. HSQC spectrum of **7** ($CDCl_3$, 400 MHz).

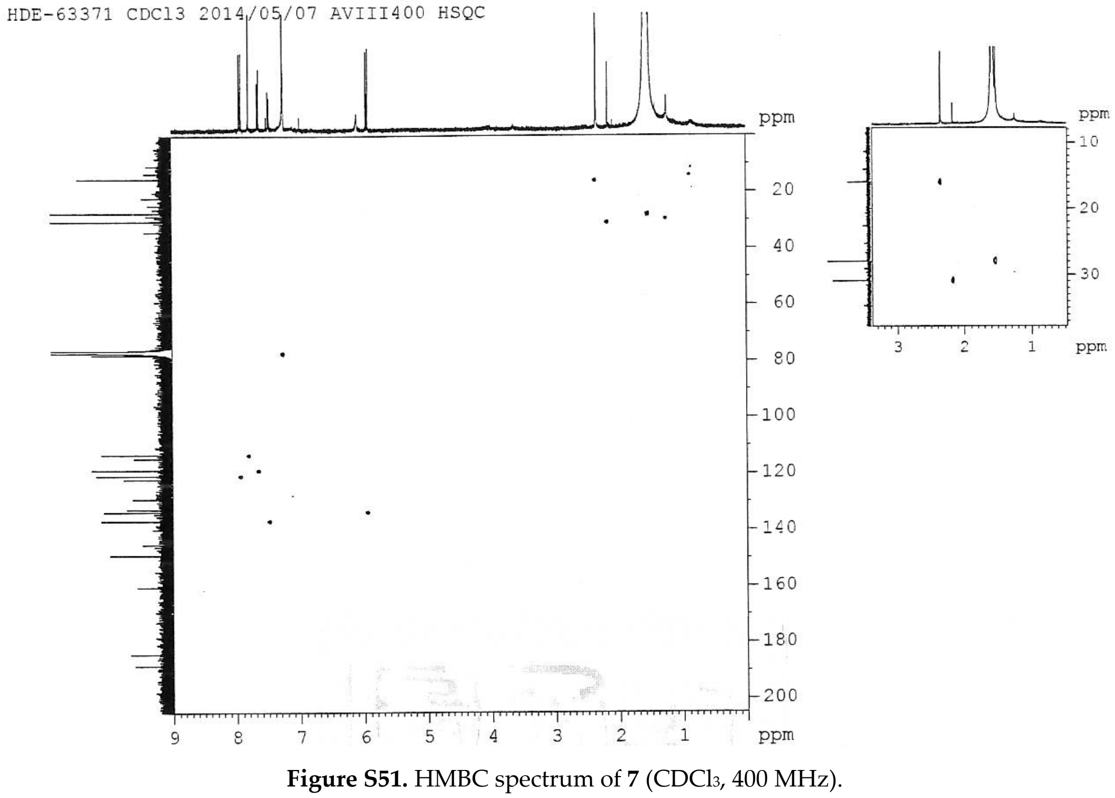

Figure S51. HMBC spectrum of **7** ($CDCl_3$, 400 MHz).

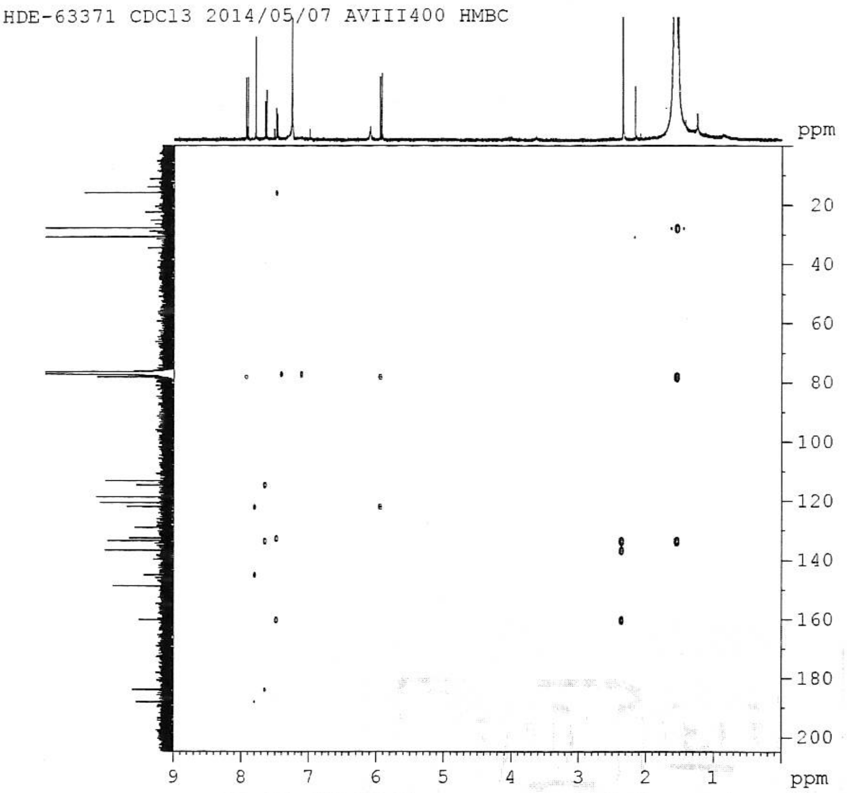

Figure S52. NOESY spectrum of **7** ($CDCl_3$, 400 MHz).

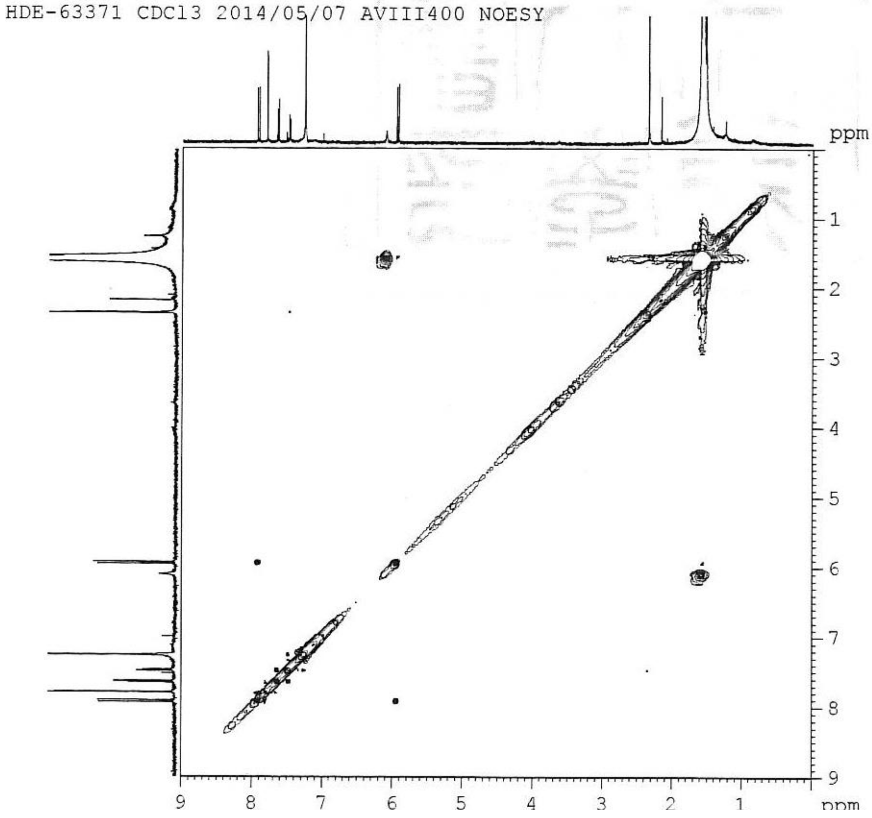

Appendix A. References for Known Compounds

2-hydroxy-6-hydroxymethylanthraquinone (**8**),[1]

tectoquinone (**9**), [2]

2-hydroxymethyl-9,10-anthraquinone (**10**), [3]

2-formyl-9,10-anthraquinone (**11**), [4]

2-hydroxy-3-methyl-9,10-anthraquinone (**12**), [5]

2-methoxy-3-methyl-9,10-anthraquinone (**13**), [6]

digiferruginol (**14**), [7]

2-hydroxy-3-hydroxymethyl-9,10-anthraquinone (**15**), [8]

1-methylalizarin (**16**), [8]

2,6-dihydroxy-3-methyl-9,10-anthraquinone (**17**), [9]

1-hydroxy-2-methoxy-3-methyl-9,10-anthraquinone (**18**), [10]

2-hydroxy-1-methoxy-3-methyl-9,10-anthraquinone (**19**), [5]

3-hydroxy-2-methoxy-6-methyl-9,10-anthraquinone (**20**), [11]

2,3-dimethoxy-6-methyl-9,10-anthraquinone (**21**), [12]

3-hydroxy-2-methoxy-6-hydroxymethyl-9,10-anthraquinone (**22**), [13]

physcion (**23**), [14]

robustaquinone B (**24**), [15]

erythroglaucin (**25**), [16]

capitellataquinone D (**26**), [17]

deacetyl asperulosidic acid methyl ester (**27**), [18]

scandoside methyl ester (**28**), [18]

E-6-*O*-*p*-coumaroyl scandoside methyl ester (**29**), [19]

Z-6-*O*-*p*-coumaroyl scandoside methyl ester (**30**), [19]

E-6-*O*-*p*-methoxycinnamoyl scandoside methyl ester (**31**), [19]

Z-6-*O*-*p*-methoxycinnamoyl scandoside methyl ester (**32**), [19]

E-6-*O*-feruloyl scandoside methyl ester (**33**), [19]

Z-6-*O*-feruloyl scandoside methyl ester (**34**), [19]

4,7-dimethoxy-5-methyl-1,3-benzodioxole (**35**), [20]

p-coumaric acid (**36**), [21]

mixture of ursolic acid (**37**) and oleanolic acid (**38**), [22]

mixture of stigmasterol (**39**) and β-sitosterol (**40**), [23]

aurantiamide acetate (**41**), [24]

References

1. Li, C. K.; Su, X. M.; Li, F. H.; Fu, J.; Wang, H. Q.; Li, B. M.; Chen, R. Y.; Kang J., Cytotoxic quinones from the aerial parts of *Morinda umbellata* L. *Phytochemistry* **2019**, *167*, 112096.
2. Endale, M.; Ekberg, A.; Alao, J. P.; Akala, H. M.; Ndakala, A.; Sunnerhagen, P.; Erdelyi, M.; Yenesew, A., Anthraquinones of the roots of *Pentas micrantha*. *Molecules* **2013**, *18*, 311-321.
3. Mitchell, D.; Lukeman, M.; Lehnherr, D.; Wan, P., Formal intramolecular photoredox chemistry of meta-substituted benzophenones. *Org. Lett.* **2005**, *7*, 3387-3389.
4. Martin, N.; Perez, I.; Sanchez, L.; Seoane, C., Synthesis and properties of the first highly conjugated tetrathiafulvalene analogues covalently attached to [60] fullerene. *J. Org. Chem.* **1997**, *62*, 5690-5695.
5. Zhang, X.; Fox, B. W.; Hadfield, J. A., Preparation of naturally occurring anthraquinones. *Synth. Commun.* **1996**, *26*, 49-62.
6. Sartori, G.; Bigi, F.; Tao, X.; Porta, C.; Maggi, R.; Predieri, G.; Lanfranchi, M.; Pellinghelli, M. A., An investigation of the reaction mechanism of the bis-acylation of aromatics with o-phthaloyl dichorides: regioselective synthesis of anthraquinones. *J. Org. Chem.* **1995**, *60*, 6588-6591.
7. Chang, P.; Lee, K. H., Cytotoxic antileukemic anthraquinones from *Morinda parvifolia*. *Phytochemistry* **1984**, *23*, 1733-1736.
8. Wu, Y. B.; Zheng, C. J.; Qin, L. P.; Sun, L. N.; Han, T.; Jiao, L.; Zhang, Q. Y.; Wu, J. Z., Antiostroporotic activity of anthraquinones from *Morinda officinalis* on osteoblasts and osteoclasts. *Molecules* **2009**, *14*, 573-583.
9. Lei, X. X.; Feng, Y. L.; Yang, S. L.; Xu, L. Z.; Li, Y. Q., A new anthraquinone from *Capparis himalayensis*. *Chem. Nat. Comp.* **2015**, *51*, 40-42.
10. Dhananjeyan, M. R.; Milev, Y. P.; Kron, M. A.; Nair, M. G., Synthesis and activity of substituted anthraquinones against a human filarial parasite, *Brugia malayi*. *J. Med. Chem.* **2005**, *48*, 2822-2830.
11. Nunez Montoya, S. C.; Agnese, A. M.; Cabrera, J. L., Anthraquinone derivatives from *Heterophyllaea pustulata*. *J. Nat. Prod.* **2006**, *69*, 801-803.
12. Punner, F.; Schieven, J.; Hilt, G., Synthesis of fluorenone and anthraquinone derivatives from aryl- and aroyl-substituted propiolates. *Org. Lett.* **2013**, *15*, 4888-4891.
13. Huang, W. H.; Yu, S. H.; Li, Y. B.; Jiang, J. Q., Two new anthraquinones from *Hedyotis diffusa*. *J. Asian Nat. Prod. Res.* **2008**, *10*, 467-471.
14. Mekkado, M.; Madrid, A.; Pena-Cortes, H.; Lopez, R.; Jara, C.; Espinoza, L., Antioxidant activity of anthraquinones isolation from leaves of *Muehlenbeckia hastulata* (J.E. SM.) johnst. (Polygonaceae). *J. Chil. Chem. Soc.* **2013**, *58*, 1767-1770.
15. Han, Y. S.; Heijden, R. V. D.; Lefeber, A. W. M.; Erkelens, C.; Verpoorte, R., Biosynthesis of anthraquinones in cell cultures of Cinchona 'Robusta' proceeds via the methylerythritol 4-phosphate pathway. *Phytochemistry* **2002**, *59*, 45-55.
16. Keller, G.; Steglich, W., 4-Aminophyscion, an anthraquinone derivative from dermocybe (Aqaricales). *Phytochemistry* **1987**, *26*, 2119-2121.
17. Ahmad, R.; Shaari, K.; Lajis, N. H.; Hamzah, A. S.; Ismail, N. H.; Kitajima, M., Anthraquinones from *Hedyotis capitellata*. *Phytochemistry* **2005**, *66*, 1141-1147.
18. Otsuka, H.; Yoshimura, K.; Yamasaki, K.; Cantoria, M. C., Isolation of 10-O-acyl iridoid glucosides from a Philippine medicinal plant, *Oldenlandia corymbosa* L. (Rubiaceae). *Chem. Pharm. Bull.* **1991**, *39*, 2049-2052.
19. Wu, H.; Tao, X.; Chen, Q.; Lao, X., Iridoids from *Hedyotis diffusa*. *J. Nat. Prod.* **1991**, *54*, 254-256.
20. Chen, P. Y.; Wu, J. D.; Tang, K. Y.; Yu, C. C.; Kuo, Y. H.; Zhong, W. B.; Lee, C. K., Isolation and synthesis of a bioactive benzenoid derivative from the fruiting bodies of *Antrodia camphorate*. *Molecules* **2013**, *18*, 7600-7608.
21. Liao, C. R.; Kuo, Y. H.; Ho, Y. L.; Wang, C. Y.; Yang, C.; Lin, C. W.; Chang, Y. S., Studies on cytotoxic constituents from the leaves of *Elaeagnus oldhamii* Maxim, in non-small cell lung cancer A549 cells. *Molecules* **2014**, *19*, 9515-9534.
22. Uddin, G.; Waliullah, B. S. S.; Alam, M.; Sadat, A.; Ahmad, A.; Uddin, A., Chemical constituents and phytotoxicity of solvent extracted fractions of stem bark of *Grewia optiva* Drummond ex Burret. *Middle East J. Sci. Res.* **2011**, *8*, 85-91.
23. Chaturvedula, V. S. P.; Prakash, I., Isolation of stigmasterol and β-sitosterol from dichloromethane extract of *Rubus suavissimus*. *Int. Curr. Pharm. J.* **2012**, *1*, 239-242.
24. Songue, J. L.; Kouam; Dongo, E.; Mpondo, T. N.; White, R. L., Chemical constituents from stem bark and roots of *Clausena anisata*. *Molecules* **2012**, *17*, 13673-13686.
